# Cathepsin B and D deficiency in the mouse pancreas induces impaired autophagy and chronic pancreatitis

**DOI:** 10.1038/s41598-021-85898-9

**Published:** 2021-03-23

**Authors:** Hideaki Iwama, Sally Mehanna, Mai Imasaka, Shinsuke Hashidume, Hiroshi Nishiura, Ken-ichi Yamamura, Chigure Suzuki, Yasuo Uchiyama, Etsuro Hatano, Masaki Ohmuraya

**Affiliations:** 1 Department of Genetics, Hyogo College of Medicine, 1-1, Mukogawa-cho, Nishinomiya, Hyogo 663-8501 Japan; 2 Department of Gastroenterological Surgery, Hyogo College of Medicine, Nishinomiya, Hyogo 663-8501 Japan; 3 Institute of Resource Development and Analysis, Kumamoto University, 2-2-1 Honjo, Chuo-ku, Kumamoto, 860-0811 Japan; 4 Division of Functional Pathology, Department of Pathology, Hyogo College of Medicine, Nishinomiya, Hyogo 663-8501 Japan; 5 Department of Pharmacology, Juntendo University Graduate School of Medicine, 2-1-1 Hongo, Bunkyo-ku, Tokyo, 113-8421 Japan; 6 Department of Cell Biology and Neuroscience, Juntendo University Graduate School of Medicine, 2-1-1 Hongo, Bunkyo-ku, Tokyo, 113-8421 Japan; 7 Present Address: Department of Veterinary Hygiene and Management, Faculty of Veterinary Medicine, Cairo University, Giza, 12211 Egypt

**Keywords:** Pancreatitis, Autophagy

## Abstract

The major lysosomal proteases, Cathepsin B (CTSB), Cathepsin D (CTSD) and Cathepsin L (CTSL), are implicated in autophagic activity. To investigate the role of each cathepsin in the exocrine pancreas, we generated mice in which the pancreas was specifically deficient in *Ctsb, Ctsd* and *Ctsl*. Each of these gene knockout (KO) and *Ctsb;Ctsl* and *Ctsd;Ctsl* double-knockout (DKO) mice were almost normal. However, we found cytoplasmic degeneration in the pancreatic acinar cells of *Ctsb;Ctsd* DKO mice, similar to autophagy related 5 (*Atg5*) KO mice. LC3 and p62 (autophagy markers) showed remarkable accumulation and the numbers of autophagosomes and autolysosomes were increased in the pancreatic acinar cells of *Ctsb;Ctsd* DKO mice. Moreover, these *Ctsb;Ctsd* DKO mice also developed chronic pancreatitis (CP). Thus, we conclude that both *Ctsb* and *Ctsd* deficiency caused impaired autophagy in the pancreatic acinar cells, and induced CP in mice.

## Introduction

Autophagy is a system of intracellular degradation that involves lysosomal enzymes. The role of autophagy is balancing sources of energy at critical times in development and in response to nutrient stress^1^. Moreover, autophagy plays a critical role in clearing misfolded or aggregated proteins, removing damaged organelles, such as mitochondria, endoplasmic reticulum and peroxisomes^1^. Autophagy exerts devastating effects in pancreatic acinar cells by the activation of trypsinogen to trypsin in the early stage of acute pancreatitis^2^. However, basal autophagy maintains pancreatic acinar cell homeostasis and protein synthesis^3^ and impaired autophagy induces chronic pancreatitis (CP)^4–7^.

Cathepsin B (CTSB) and L (CTSL) are cysteine proteases, and Cathepsin D (CTSD) is an aspartic protease^8^. These cathepsins are major lysosomal proteases and are widely expressed in endosomes and lysosomes. Mice lacking *Ctsd* in all tissues die one month after birth due to intestinal necrosis^9^. Mice lacking *Ctsb* or *Ctsl* in all tissues grow up almost normally^10–12^, however, mice lacking both *Ctsb* and *Ctsl* in all tissues die 2 weeks after birth due to the massive apoptosis of central neurons^13^. Thus, the organ-specific roles of these cathepsins are different. Furthermore, Ctsb and Ctsl were reported to be involved in the processing of CtsD^14,15^, and cathepsins might interact with each other in different ways depending on tissues^16,17^.

On the other hand, Ctsb and Ctsl play a role in intrapancreatic trypsinogen activation and the onset of acute pancreatitis^18–20^. Ctsd was reported to regulate Ctsb activation during experimental pancreatitis^17^, and was implicated in Ctsb and Ctsl degradation, but not in autophagic activity in the pancreas^21^. Thus, it is not clear which of the cathepsins plays an important role in autophagy of the pancreas.

In order to investigate the role of Ctsb, Ctsd and Ctsl in autophagy of the pancreas, we generated mice in which the pancreas was specifically deficient in *Ctsb, Ctsd, Ctsl, Ctsb;Ctsd, Ctsb;Ctsl* or *Ctsd;Ctsl*. The purpose of this study was to clarify which of these cathepsins are essential for autophagy in the mouse pancreas.

## Results

### Generation of pancreas-specific *Ctsb-, Ctsd-, and Ctsl-*deficient mice

**Figure 1:**
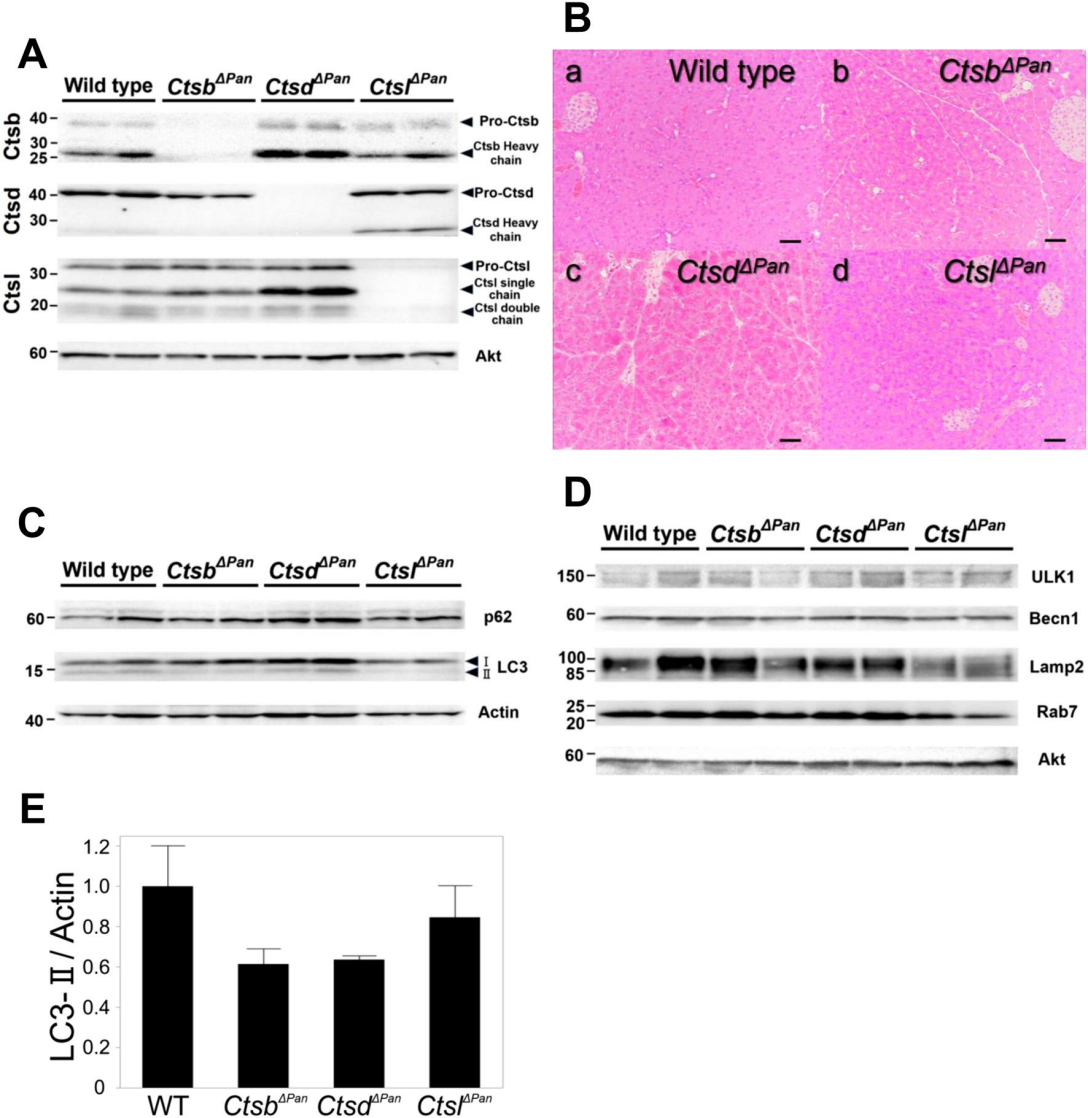
Generation of *Ctsb*^*ΔPan*^, *Ctsd*^*ΔPan*^ and *Ctsl*^*ΔPan*^ mice. (**A**) Western blotting of Ctsb, Ctsd and Ctsl in the pancreases of 1-month-old wild-type, *Ctsb*^*ΔPan*^, *Ctsd*^*ΔPan*^ and *Ctsl*^*ΔPan*^ mice during normal feeding. The full-length blots/gels and the quantification data of WB are presented in Supplementary figure S1–S4. (**B**) HE staining of pancreas tissue isolated from a 2-month-old wild-type (a), *Ctsb*^*ΔPan*^ (b), *Ctsd*^*ΔPan*^ (c) and *Ctsl*^*ΔPan*^ (d) mice during normal feeding. Scale bars indicate 50 μm. (**C**) Western blotting of pancreas extracts with anti-p62 and anti-LC3 antibodies in 1-month-old wild-type, *Ctsb*^*ΔPan*^, *Ctsd*^*ΔPan*^ and *Ctsl*^*ΔPan*^ mice during normal feeding. Actin was used as a loading control. The full-length blots/gels and the quantification data of WB are presented in supplementary Figure S5–S7. (**D**) Western blotting of pancreas extracts with anti-ULK1, anti-Becn1, anti-Lamp2 and anti-Rab7 antibodies in 1-month-old wild-type, *Ctsb*^*ΔPan*^, *Ctsd*^*ΔPan*^ and *Ctsl*^*ΔPan*^ mice during normal feeding. Akt was used as a loading control. The full-length blots/gels and the quantification data of WB are presented in supplementary figure S8–S12. (**E**) The ratio of LC3-II/Actin in the pancreases of 1-month-old wild-type, *Ctsb*^*ΔPan*^, *Ctsd*^*ΔPan*^ and *Ctsl*^*ΔPan*^ mice during normal feeding. Results are shown as the mean ± SEM. n = 3 mice per condition. There was no apparent difference in the ratio of LC3-II/Actin among these animals.

**Figure 2:**
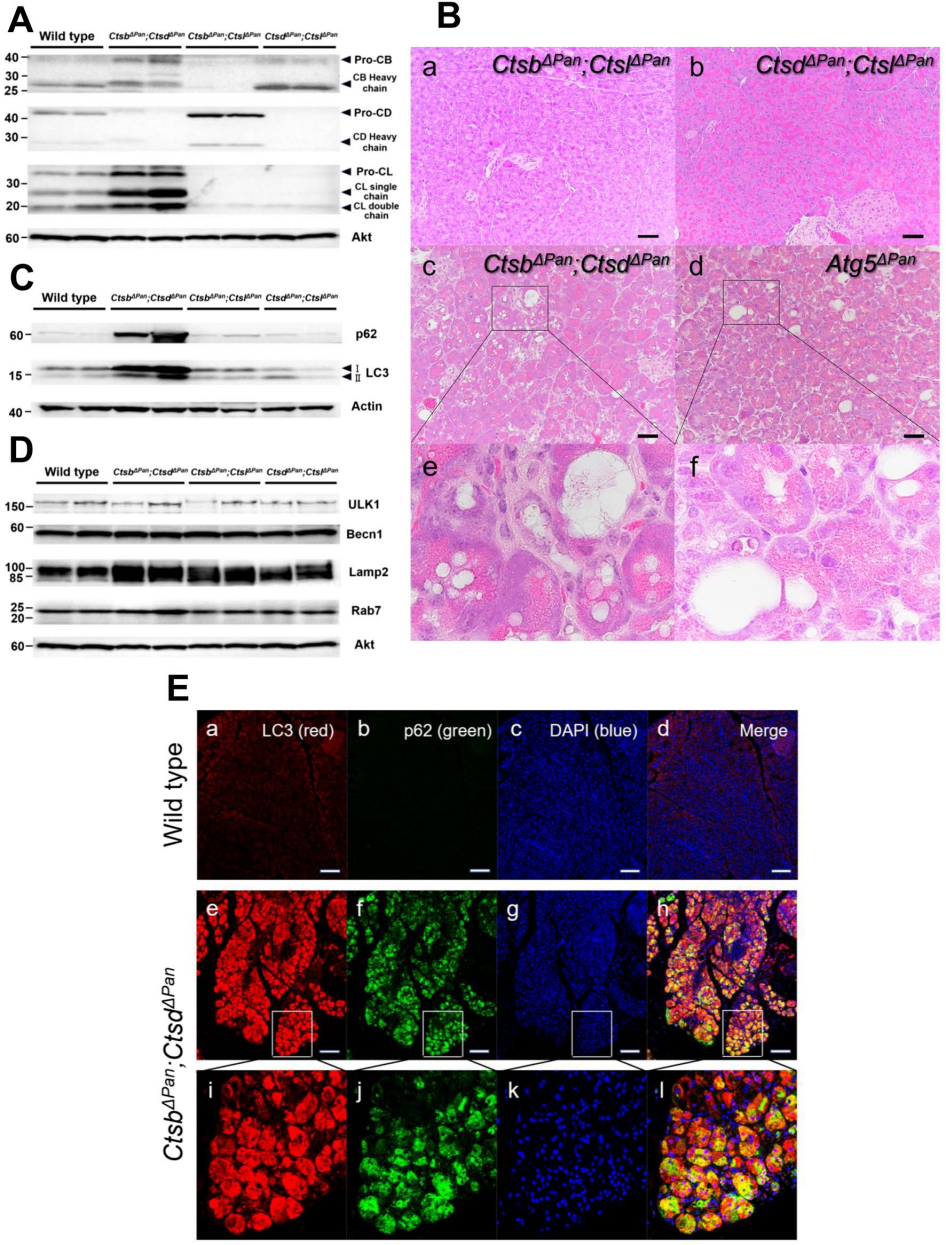
Generation of *Ctsb*^*ΔPan*^*;Ctsd*^*ΔPan*^, *Ctsb*^*ΔPan*^*;Ctsl*^*ΔPan*^ and *Ctsd*^*ΔPan*^*;Ctsl*^*ΔPan*^ mice. (**A**) Western blotting of Ctsb, Ctsd and Ctsl in the pancreases of 1-month-old wild-type, *Ctsb*^*ΔPan*^*;Ctsd*^*ΔPan*^, *Ctsb*^*ΔPan*^*;Ctsl*^*ΔPan*^ and *Ctsd*^*ΔPan*^*;Ctsl*^*ΔPan*^ mice during normal feeding. The full-length blots/gels and the quantification data of WB are presented in supplementary figure S13–S16. (**B**) HE staining of pancreas tissue isolated from 2-month-old *Ctsb*^*ΔPan*^*;Ctsd*^*ΔPan*^, *Ctsb*^*ΔPan*^*;Ctsl*^*ΔPan*^, *Ctsd*^*ΔPan*^*;Ctsl*^*ΔPan*^ and *Atg5*^*ΔPan*^ mice during normal feeding. Scale bars indicate 50 μm. (**C**) Western blotting of pancreas extracts with anti-p62 and anti-LC3 antibodies in 1-month-old wild-type, *Ctsb*^*ΔPan*^*;Ctsd*^*ΔPan*^, *Ctsb*^*ΔPan*^*;Ctsl*^*ΔPan*^ and *Ctsd*^*ΔPan*^*;Ctsl*^*ΔPan*^ mice during normal feeding. Actin was used as a loading control. The full-length blots/gels and the quantification data of WB are presented in supplementary figure S17–S19. (**D**) Western blotting of pancreas extracts with anti-ULK1, anti-Becn1, anti-Lamp2 and anti-Rab7 antibodies in 1-month-old wild-type, *Ctsb*^*ΔPan*^*;Ctsd*^*ΔPan*^, *Ctsb*^*ΔPan*^*;Ctsl*^*ΔPan*^ and *Ctsd*^*ΔPan*^*;Ctsl*^*ΔPan*^ mice during normal feeding. Akt was used as a loading control. The full-length blots/gels and the quantification data of WB are presented in supplementary figure S20–S24. (**E**) Immunofluorescent staining of pancreas tissue isolated from 2-month-old wild-type and *Ctsb*^*ΔPan*^*;Ctsd*^*ΔPan*^ mice during normal feeding. Antibodies against LC3 and p62 were used for immunostaining. Scale bars indicate 100 μm.

In order to investigate the role of Ctsb, Ctsd and Ctsl in autophagy of the pancreas, we generated pancreas-specific *Ctsb-, Ctsd-* or *Ctsl-*deficient (*Ctsb*^*ΔPan*^*, Ctsd*^*ΔPan*^*, Ctsl*^*ΔPan*^) mice using the Cre-*loxP* system. Pancreas-specific *Ctsb;Ctsd, Ctsb;Ctsl* and *Ctsd;Ctsl* double-knockout (DKO) (*Ctsb*^*ΔPan*^*;Ctsd*^*ΔPan*^*, Ctsb*^*ΔPan*^*;Ctsl*^*ΔPan*^ and *Ctsd*^*ΔPan*^*;Ctsl*^*ΔPan*^) mice were obtained by mating the *Ctsb*^*ΔPan*^*, Ctsd*^*ΔPan*^ and *Ctsl*^*ΔPan*^ mice. Western blotting (WB) of whole tissue lysates of the pancreases of wild-type, *Ctsb*^*ΔPan*^*, Ctsd*^*ΔPan*^ and *Ctsl*^*ΔPan*^ mice confirmed that *Ctsb*, *Ctsd* or *Ctsl* was disrupted in the *Ctsb*^*ΔPan*^*, Ctsd*^*ΔPan*^ and *Ctsl*^*ΔPan*^ pancreas, respectively (Fig. 1A). WB of whole tissue lysates of the pancreases of wild-type Fig. S1, *Ctsb*^*ΔPan*^*;Ctsd*^*ΔPan*^*, Ctsb*^*ΔPan*^*;Ctsl*^*ΔPan*^ and *Ctsd*^*ΔPan*^*;Ctsl*^*ΔPan*^ mice confirmed that *Ctsb* and *Ctsd, Ctsb* and *Ctsl* or *Ctsd* and *Ctsl* were disrupted in the *Ctsb*^*ΔPan*^*;Ctsd*^*ΔPan*^*, Ctsb*^*ΔPan*^*;Ctsl*^*ΔPan*^ and *Ctsd*^*ΔPan*^*;Ctsl*^*ΔPan*^ pancreas, respectively (Fig. 2A). The *Ctsb*^*ΔPan*^*, Ctsd*^*ΔPan*^ and *Ctsl*^*ΔPan*^ mice were all healthy, fertile, and indistinguishable from their wild-type littermates, although the expression of each of the cathepsins was completely deleted.

### The expression of autophagy markers was normal in the pancreas of *Ctsb*^*ΔPan*^*, Ctsd*^*ΔPan*^ and *Ctsl*^*ΔPan*^ mice

In order to investigate the autophagic activity in the pancreases of *Ctsb*^*ΔPan*^*, Ctsd*^*ΔPan*^ or *Ctsl*^*ΔPan*^ mice, we firstly performed histopathological examinations of the pancreases of *Ctsb*^*ΔPan*^*, Ctsd*^*ΔPan*^ and *Ctsl*^*ΔPan*^ mice. These examinations revealed no abnormalities (Fig. 1Ba–d).

We then investigated the expression of p62/sequestosome1 (p62), a selective substrate of autophagy, and microtubule-associated protein 1 light chain 3 (LC3), a marker of autophagic vacuole formation, in order to analyze the autophagy function in the pancreases of *Ctsb*^*ΔPan*^*, Ctsd*^*ΔPan*^ and *Ctsl*^*ΔPan*^ mice. As shown in Fig. 1C, no significant difference in the expression of p62 in the pancreases was noted among wild-type, *Ctsb*^*ΔPan*^*, Ctsd*^*ΔPan*^ and *Ctsl*^*ΔPan*^ mice (Fig. 1C). LC3 has two forms. LC3-I is localized in the cytoplasm and is converted into LC3-II. LC3-II is associated with the membranes of autophagosomes in the phosphatidylethanolamine conjugated form, and the amount of LC3-II is correlated with the extent of autophagosome formation. In the pancreases of wild-type, *Ctsb*^*ΔPan*^*, Ctsd*^*ΔPan*^ and *Ctsl*^*ΔPan*^ mice, there was no apparent difference in the ratio of LC3-II/Actin (Fig. 1E).

We next examined the expression of UNC-51-like kinase 1 (ULK1), Beclin1 (Becn1), lysosomal-associated membrane protein 2 (Lamp2) and Rab7, a member of the Ras-related GTP-binding protein family, by WB. ULK1 interfaces with mammalian target of rapamycin complex 1 (mTORC1)^22^, and Becn1 forms a regulatory complex with class III phosphatidylinositol-3-kinase^22^. In this way, both act in the phase of autophagy induction. Lamp2 is a lysosome-associated membrane protein^7^ and Rab7 functions in various intracellular vesicle trafficking systems including autophagy and endocytosis^23^. Thus, Lamp2 and Rab7 are associated with the formation of autolysosomes. Among the pancreases of wild-type, *Ctsb*^*ΔPan*^*, Ctsd*^*ΔPan*^ and *Ctsl*^*ΔPan*^ mice, there were no apparent differences in the expression of ULK1, Becn1, Lamp2, or Rab7 (Fig. 1D). These data indicate that autophagy was not impaired in the pancreases of *Ctsb*^*ΔPan*^*, Ctsd*^*ΔPan*^ or *Ctsl*^*ΔPan*^ mice.

### Impaired autophagy was induced in the pancreas of *Ctsb*^*ΔPan*^*;Ctsd*^*ΔPan*^ mice

In order to investigate the autophagic activity in the pancreases of *Ctsb*^*ΔPan*^*;Ctsd*^*ΔPan*^*, Ctsb*^*ΔPan*^*;Ctsl*^*ΔPan*^ and *Ctsd*^*ΔPan*^*;Ctsl*^*ΔPan*^ mice, we performed histopathological examinations of the pancreases of *Ctsb*^*ΔPan*^*;Ctsd*^*ΔPan*^*, Ctsb*^*ΔPan*^*;Ctsl*^*ΔPan*^ and *Ctsd*^*ΔPan*^*;Ctsl*^*ΔPan*^ mice and detected no abnormalities in the pancreases of *Ctsb*^*ΔPan*^*;Ctsl*^*ΔPan*^ and *Ctsd*^*ΔPan*^*;Ctsl*^*ΔPan*^ mice (Fig. 2Ba, b). On the other hand, the acinar cells in the pancreas of *Ctsb*^*ΔPan*^*;Ctsd*^*ΔPan*^ mice exhibited on cytoplasmic vacuolization (Fig. 2Bc, e), which was similar to the mice with pancreas-specific disruption of autophagy related 5 (*Atg5*) (*Atg5*^*ΔPan*^) (Fig. 2Bd, f).

We then investigated the expression of p62 and LC3 in order to analyze the autophagy function in the pancreases of *Ctsb*^*ΔPan*^*;Ctsd*^*ΔPan*^*, Ctsb*^*ΔPan*^*;Ctsl*^*ΔPan*^ or *Ctsd*^*ΔPan*^*;Ctsl*^*ΔPan*^ mice. As shown in Fig. 2C, there were no significant differences in the expressions of p62 and LC3 among wild-type, *Ctsb*^*ΔPan*^*;Ctsl*^*ΔPan*^ and *Ctsd*^*ΔPan*^*;Ctsl*^*ΔPan*^ mice. On the other hand, *Ctsb*^*ΔPan*^*;Ctsd*^*ΔPan*^ mice showed a marked increase in the autophagy substrates p62 and the membrane-bound form of LC3 (Fig. 2C). Furthermore, we performed immunofluorescent staining of the pancreases of wild-type and *Ctsb*^*ΔPan*^*;Ctsd*^*ΔPan*^ mice, which demonstrated the accumulation of p62 and LC3 in the acinar cells of *Ctsb*^*ΔPan*^*;Ctsd*^*ΔPan*^ mice (Fig. 2Ea–l).

We also examined the expression of ULK1, Becn1, Lamp2 and Rab7 by WB. The expression of ULK1, Becn1, Lamp2 and Rab7 in wild-type, *Ctsb*^*ΔPan*^*;Ctsd*^*ΔPan*^*, Ctsb*^*ΔPan*^*;Ctsl*^*ΔPan*^ and *Ctsd*^*ΔPan*^*;Ctsl*^*ΔPan*^ mice showed no apparent difference (Fig. 2D). These data indicate that autophagy was impaired in the pancreas of *Ctsb*^*ΔPan*^*;Ctsd*^*ΔPan*^ mice.

### Autolysosomes accumulated in pancreatic acinar cells of *Ctsb*^*ΔPan*^*;Ctsd*^*ΔPan*^ mice, while autophagosomes were also present in the cells but fewer in number

**Figure 3:**
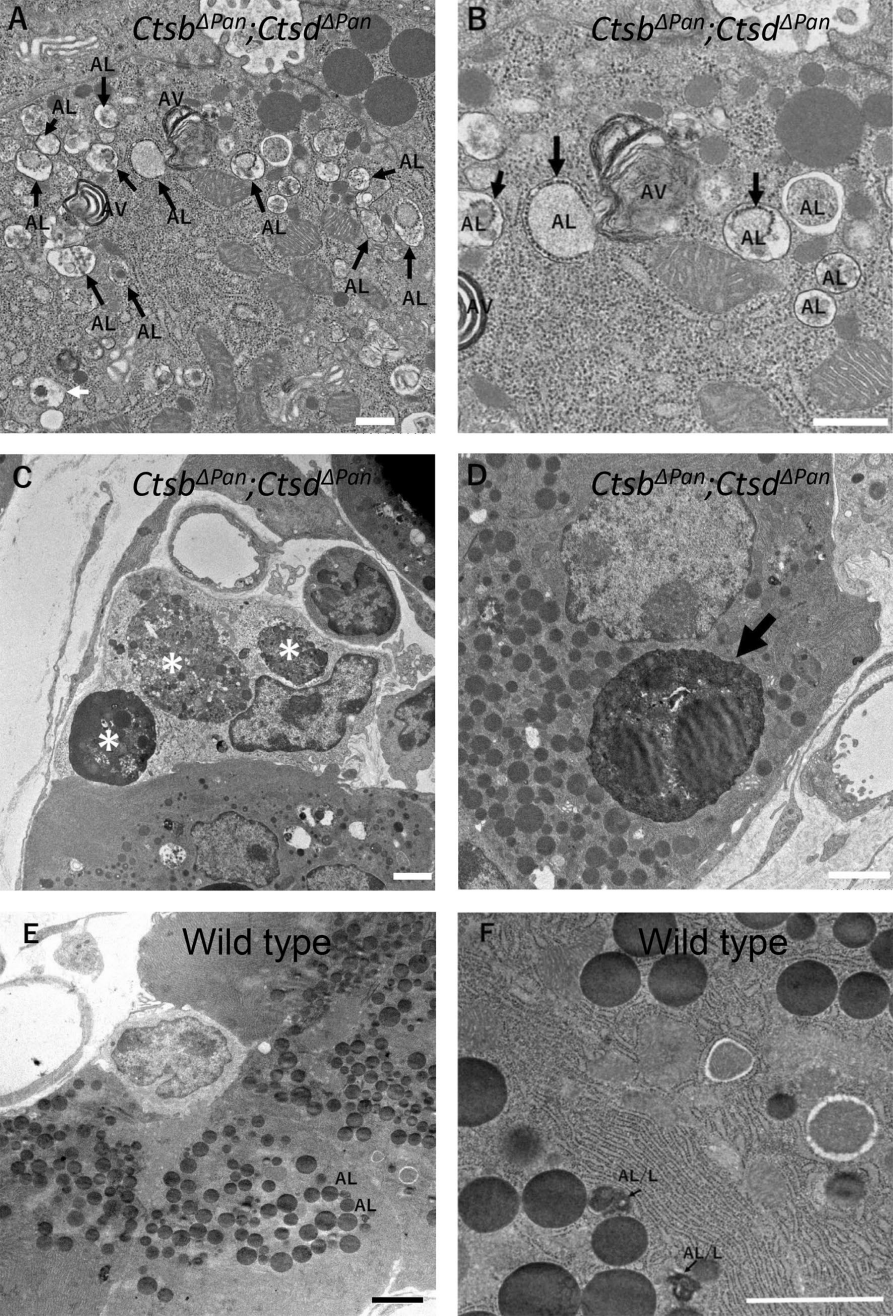
Transmission electron microscopy of wild-type and *Ctsb*^*ΔPan*^*;Ctsd*^*ΔPan*^ mice. Transmission electron microscopy of pancreases from 1-month-old wild-type and *Ctsb*^*ΔPan*^*;Ctsd*^*ΔPan*^ mice. A large number of granular autolysosome-like structures (AL) are observed in the pancreatic acinar cells of *Ctsb*^*ΔPan*^*;Ctsd*^*ΔPan*^ mice, and autophagosome-like structures (AV) can also be seen in the cells although fewer in number (**A**,**B**) than in the cells of wild-type mice (**E**,**F**). Several inclusion bodies with dying acinar cell debris are detected in a phagocytic cell appearing in the vicinity of *Ctsb*^*ΔPan*^*;Ctsd*^*ΔPan*^ mouse acinar cells as well as *Ctsb*^*ΔPan*^*;Ctsd*^*ΔPan*^ mouse acinar cells (**C**,**D**). All scale bars indicate 20 μm.

In order to clarify which phase of the autophagic pathway was disturbed in the pancreas of *Ctsb*^*ΔPan*^*;Ctsd*^*ΔPan*^ mouse pancreas, in the view of the morphology, we observed the pancreas using a transmission electron microscope (TEM). A large number of granular structures were observed in the pancreatic acinar cells of *Ctsb*^*ΔPan*^*;Ctsd*^*ΔPan*^ mice compared with wild-type mice (Fig. 3A,B,E,F). In the cytoplasm of the acinar cells of *Ctsb*^*ΔPan*^*;Ctsd*^*ΔPan*^ mice, most granular structures that possessed cytoplasmic organelles with degraded or undegraded materials, in particular the granular endoplasmic reticulum with a large amount of synthetized materials, were autolysosome-like structures (Fig. 3A,B). In addition to such autolysosome-like structures, the *Ctsb*^*ΔPan*^*;Ctsd*^*ΔPan*^ acinar cells had multi-membranous vacuolar structures that corresponded to the autophagosomes often seen in cathepsin D-defective neurons^24^ (Fig. 3A,B) but were much fewer in number. The *Ctsb*^*ΔPan*^*;Ctsd*^*ΔPan*^ phagocytic cells that appeared adjacent to acinar cells often contained large granules of cellular debris in which zymogen granules and nuclear structures were detected (Fig. 3C). In some cases, inclusion bodies which contained a nucleus with condensed chromatin were detected in acinar cells (Fig. 3D). In contrast, wild-type acinar cells possessed lysosome/autolysosome-like structures in the cytoplasm, but no clear-cut autophagosome-like structures were detected in the cells, in contrast to *Ctsb*^*ΔPan*^*;Ctsd*^*ΔPan*^ acinar cells (Fig. 3E,F).

### Chronic pancreatitis was induced in *Ctsb*^*ΔPan*^*;Ctsd*^*ΔPan*^ mice

**Figure 4:**
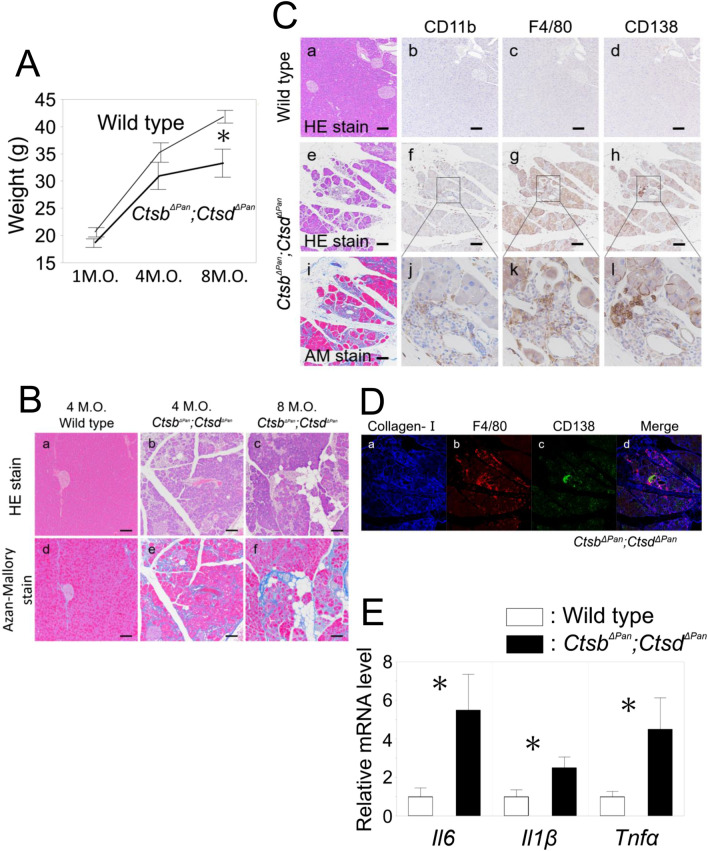
Chronic pancreatitis was induced in *Ctsb*^*ΔPan*^*;Ctsd*^*ΔPan*^ mice. (**A**) Body weights of 1-, 4- and 8-month-old wild-type and *Ctsb*^*ΔPan*^*;Ctsd*^*ΔPan*^ mice (n = 8–15 Wild type, n = 5–8 *Ctsb*^*ΔPan*^*;Ctsd*^*ΔPan*^). *P < 0.01. M.O.: month-old. (**B**) HE staining and Azan-Mallory staining of pancreas tissue isolated from 4-month-old wild-type (a, d) and *Ctsb*^*ΔPan*^*;Ctsd*^*ΔPan*^ (b, e) mice, and 8-month-old *Ctsb*^*ΔPan*^*;Ctsd*^*ΔPan*^ (c, f) mice during normal feeding. Scale bars indicate 50 μm. AM: Azan-Mallory. (**C**) The Immunohistochemical analysis of pancreas tissue isolated from 4-month-old wild-type (a–d) and *Ctsb*^*ΔPan*^*;Ctsd*^*ΔPan*^ (e–l) mice. Antibodies against CD11b as a monocyte marker, F4/80 as a microphage marker and CD138 as a plasma cell marker were used for immunostaining. Scale bars indicate 50 μm. (**D**) Immunofluorescent staining of pancreas tissue isolated from 4-month-old *Ctsb*^*ΔPan*^*;Ctsd*^*ΔPan*^ mice (a–d) during normal feeding. Antibodies aginst Collagen-I as a marker of fibrosis, and F4/80 and CD138 as a marker of inflammatory cell were used for immunostaining. (**E**) qRT-PCR of pancreatic RNAs from 2-month-old wild-type and *Ctsb*^*ΔPan*^*;Ctsd*^*ΔPan*^ mice for cytokine genes. Results are shown as the mean ± SEM. n = 6 mice per condition. *P < 0.05.

We compared the weight at one, four and eight months old between wild-type mice and *Ctsb*^*ΔPan*^*;Ctsd*^*ΔPan*^ mice. No significant difference was noted between the weights at one and four months old, but the weight at eight months old was significantly lower in *Ctsb*^*ΔPan*^*;Ctsd*^*ΔPan*^ mice than in wild-type mice (Fig. 4A).

On a histopathologic examination of the pancreas in four-month-old *Ctsb*^*ΔPan*^*;Ctsd*^*ΔPan*^ mice, numerous vacuoles were observed in the acinar cells, in comparison to wild-type mice (Fig. 4Ba, b, d). The loss of the normal architecture and replacement of acinar cells with fat was observed in eight-month-old *Ctsb*^*ΔPan*^*;Ctsd*^*ΔPan*^ mice (Fig. 4Bc). Pancreatic fibrosis in the pancreases of four-month-old and eight-month-old *Ctsb*^*ΔPan*^*;Ctsd*^*ΔPan*^ mice was confirmed by Azan-Mallory staining (Fig. 4Be, f, Ci). Fibrosis and replacement of acinar cells with fat worsened over time in the pancreas of eight-month-old *Ctsb*^*ΔPan*^*;Ctsd*^*ΔPan*^ mice compared with the pancreas of four-month-old *Ctsb*^*ΔPan*^*;Ctsd*^*ΔPan*^ mice. Immunostaining of the pancreases in four-month-old *Ctsb*^*ΔPan*^*;Ctsd*^*ΔPan*^ mice revealed a large number of inflammatory cells, including monocytes (CD11b-positive cells), macrophages (F4/80-positive cells) and plasma cells (CD138-positive cells) infiltrated the pancreas (Fig. 4Ce–h, j–l). No monocyte, macrophage nor plasma cell was detected in the pancreases of four-month-old wild-type mice (Fig. 4Ca–d). Immunofluorescent staining of the pancreas in four-month-old mice showed chronic inflammatory cell infiltration and progressive fibrosis (Fig. 4D).

The expression of mRNAs encoding proinflammatory cytokines, including *interleukin 6* (*Il6*), *interleukin 1β* (*Il1β*) and *tumor necrosis factor α* (*Tnfα*), was also upregulated in *Ctsb*^*ΔPan*^*;Ctsd*^*ΔPan*^ mice (Fig. 4E). These data suggest that CP was induced in the *Ctsb*^*ΔPan*^*;Ctsd*^*ΔPan*^ mouse pancreas.

## Discussion

The purpose of this study is to clarify the role of cathepsin B, D and L in autophagy of the pancreas. The pancreas of *Ctsb*^*ΔPan*^*;Ctsd*^*ΔPan*^ mice showed impaired autophagy. On the other hand, autophagy was not impaired in the pancreases of *Ctsb*^*ΔPan*^ and *Ctsd*^*ΔPan*^ mice. These results indicate that Ctsb and Ctsd play an important role and act synergistically in the autophagy of the pancreas.

Ctsd plays a critical role in the autophagy of the intestine^9^ and the autophagy of central nerves requires Ctsb or Ctsl^13^. This study proved that the autophagy of pancreatic acinar cells in mice required both Ctsb and Ctsd. In this study, the autophagy in the pancreas was not impaired in *Ctsb*^*ΔPan*^*, Ctsd*^*ΔPan*^ or *Ctsl*^*ΔPan*^ mice, as reported previously^10–12,21^. The Ctsb and Ctsl expression was increased in the pancreas of *Ctsd*^*ΔPan*^ mice. The same result was reported previously^21^. In this study, the expression of LC3-II was not increased in the pancreas of *Ctsd*^*ΔPan*^ mice. A previous report^21^ evaluated the ratio of LC3-II to LC3-I. LC3-II should be calculated using the ratio of a housekeeping protein, not LC3-I, as LC3-I tends to be less sensitive to detection by certain anti-LC3 antibodies^25^. This is the reason why we calculated the ratio of LC3-II to actin. In addition, the serine protease inhibitor Kazal type 3 (*Spink3*), which is a trypsin specific inhibitor in the pancreas*, -cre* knock-in mice were used in the previous report^21^, while pancreas transcription factor 1 subunit alpha (*Ptf1a*) *-cre* knock-in mice (*Ptf1a*^*cre/*+^) were used in this study. *Spink3* expression was reported in not only the pancreas, but also in the intestine, kidney and epididymis, among other sites^26^. *Ptf1a* expression would be more specific to the pancreas than *Spink3*, so we used *Ptf1a*^*cre/*+^ mice in this study.

The expression of Ctsb in *Ctsb*^*ΔPan*^*;Ctsd*^*ΔPan*^ mice did not disappeared. However, the expression ratio of pro-Ctsb/Ctsb heavy chain in *Ctsb*^*ΔPan*^*;Ctsd*^*ΔPan*^ mice was different from in wild-type mice. Ctsd in pancreatic acinar cells was shown to be involved in Ctsb and Ctsl degradation^21^ and regulated Ctsb activation under conditions of experimental pancreatitis^17^. Ctsd was shown to be accumulated in the brain of *Ctsb;Ctsl* DKO mice in all cells^13^. These results indicate that Ctsb, Ctsd and Ctsl regulate each other in vivo. The *Ctsb*^*ΔPan*^*;Ctsd*^*ΔPan*^ mice showed a marked increase in the autophagy substrates p62 and LC3. This result indicates that impaired autophagy was induced in the *Ctsb*^*ΔPan*^*;Ctsd*^*ΔPan*^ mouse pancreas. Because Ctsb and Ctsd are lysosomal enzymes, autophagy in the *Ctsb*^*ΔPan*^*;Ctsd*^*ΔPan*^ mouse pancreas could be disturbed in the degradation phase in autolysosomes. The accumulation of autolysosomes with a few autophagosomes in the pancreatic acinar cells of *Ctsb*^*ΔPan*^*;Ctsd*^*ΔPan*^ mice was consistent with this expectation.

In the histopathologic examination, the *Ctsb*^*ΔPan*^*;Ctsd*^*ΔPan*^ mouse pancreas showed CP, which progressed with time. At least four animal models of CP with impaired autophagy have been previously reported: mice with pancreas-specific disruption of *Atg5* that developed a form of CP^4^, mice with pancreas-specific ablation of IκB kinase α^5^, mice lacking *Spink3* with a mosaic pattern of *SPINK1* expression^6^, and LAMP-2-deficient mice^7^. The histological findings of CP, such as inflammation, acinar-to-ductal metaplasia and acinar-cell hypertrophy, have been confirmed in all these models. Additional experiments have been performed, such as qRT-PCR analyses of pancreatic RNA from fibrogenic markers and cytokine and chemokine genes and immune cell markers, assessments of serum levels of pancreatitis markers, evaluations of changes in the body and pancreas weight and immunostaining of the pancreas. On the other hand, impaired autophagy was evidenced by accumulation of p62 in WB, enlarged autophagic vacuoles in HE staining and TEM of pancreatic acinar cells. Immunostaining of the pancreas was also performed as an additional experiment. *Ctsb*^*ΔPan*^*;Ctsd*^*ΔPan*^ mice may be another animal model of CP with impaired autophagy.

In 1896, Chiari reported that pancreatitis was the result of pancreatic autodigestion^27,28^. In 1959, Greenbaum reported Ctsb activated trypsinogen to trypsin in vitro^29^. Steer suggested that Ctsb might be responsible for the intracellular activation of digestive enzymes^30–32^. It became clear that Ctsb inhibition reduced the severity of pancreatitis^33^, the release of Ctsb in cytosol caused cell death in acute pancreatitis^34^, and Ctsb activity initiated experimental pancreatitis^35^. Ctsb, as described above, is known to be deeply implicated in pancreatitis. The present study also indicated that Ctsb played an important role of autophagy in pancreas. Ctsd has many functions as an aspartic protease^36–38^, that interacts with other important molecules and influences cell signals^39–41^. Ctsd is implicated in apoptosis and cancer, but not in the severity of pancreatitis or autophagy activity by itself^21^. From the above results, both Ctsb and Ctsd play an important synergistic role in the autophagy of the mouse pancreas.

In conclusion, both *Ctsb* and *Ctsd* deficiency caused impaired autophagy in pancreatic acinar cells and induced CP in mice. A future challenge is to clarify the mechanism underlying the interaction of Ctsb and Ctsd with regard to autophagy.

## Materials and methods

### Animal protocol and experimental design

Mice were kept under specific-pathogen-free conditions with ad libitum access to food and water in a 12 h (h) light/dark cycle. C57BL/6 N mice were purchased from CREA Japan. All animal experiments were performed with the approval of the Hyogo College of Medicine Institutional Animal Care and Use Committee (18–068), and the ARRIVE guideline. All methods were carried out in accordance with the relevant guidelines of the Hyogo College of Medicine and the ARRIVE guideline, including any relevant details.

### Generation of *Ctsb, Ctsd, Ctsl, Ctsb;Ctsd, Ctsb;Ctsl* and *Ctsd;Ctsl* deficient mice in the pancreas

We generated mice where the third exon of *Ctsb*, the second exon of *Ctsd* or the third to sixth exon of *Ctsl* was flanked by *loxP* sites. Both *Ctsb*^*f/*+^ and *Ctsl*^*f/*+^ ES cells were purchased from the International Mouse Phenotyping Consortium (IMPC) project. *Ctsd*^*f/*+^ are previously described^21^. Mice homozygous for these modifications are denoted by *Ctsb*^*f/f*^, *Ctsd*^*f/f*^ or *Ctsl*^*f/f*^. *Ctsb*^*f/f*^, *Ctsd*^*f/f*^ or *Ctsl*^*f/f*^ mice were crossed with *Ptf1a*^*cre/*+^ mice^42^ and offspring carrying *Ptf1a*^*cre/*+^ and two copies of the floxed *Ctsb*, *Ctsd* or *Ctsl* allele (*Ctsb*^*f/f*^*;Ptf1a*^*cre/*+^, *Ctsd*^*f/f*^*;Ptf1a*^*cre/*+^ or *Ctsl*^*f/f*^*;Ptf1a*^*cre/*+^) were used in this study as a homozygous mutant (*Ctsb*^*ΔPan*^, *Ctsd*^*ΔPan*^ or *Ctsl*^*ΔPan*^) mice. The *Ctsb;Ctsd, Ctsb;Ctsl* or *Ctsd;Ctsl* DKO mice were obtained by mating the *Ctsb*^*ΔPan*^*, Ctsd*^*ΔPan*^ and *Ctsl*^*ΔPan*^ mice.

### Antibodies

Antibodies against cathepsin B (R&D Systems, AF965), cathepsin D (Santa Cruz, sc-6486), cathepsin L (R&B Systems, AF1515), p62 (MBL, PM045), LC3 (Cell Signaling Technology, 2775 and 43566S), ULK1 (Sigma, A7481), LAMP2 (Sigma, L0668), Rab7 (Sigma, R8779), Becn1 (Santa Cruz, 11427), Akt (Cell Signaling Technology, 9272), Actin (Sigma, A5060), F4/80 (AbD Serotec, MCAP497), CD11b (Abcam, ab133357), CD138 (BD Biosciences, 553712) and Collagen- I (Abcam, ab34710) were used as primary antibodies in this study. The secondary antibodies used in this study were anti-rabbit (GE Healthcare, NA9340) (Jackson ImmunoResearch, 712-035-153 and 711-035-152), anti-mouse (Jackson ImmunoResearch, 115-035-146) and anti-goat (CHEMICON INTERNATIONAL, AP106P).

### Histological and immunohistochemical analyses

For the histological analyses, pancreatic tissue was fixed overnight in 15% formalin, embedded in paraffin, sectioned, and subjected to HE and Azan-Mallory staining. Immunohistochemistry was performed using the antibodies listed above.

### Immunofluorescent staining with confocal microscopy

For multiple immunofluorescent immunostaining, tissue samples were fixed in 15% buffered formalin and embedded in paraffin. In order to investigate fibrosis and inflammatory cells of macrophages and plasma cells, triple fluorescent immunostaining was performed with antibodies against Collagen-I, F4/80 and CD138. Subsequent staining was performed using an Opal 4-color IHC kit (Akoya Biosciences NEL820001KT). Signal amplification and covalent binding of fluorophore was achieved using a tyramide signaling amplification reagent (included in the Opal kit) that is conjugated with a different fluorophore for each cycle. Tissue samples were incubated overnight at 4 °C with the primary antibody, as follows: LC3(1:1500; detected by Opal 570 at 1:300), p62 (1:15,000; detected by Opal 520 at 1:300), Collagen-I (1:1200; detected by Opal 690 at 1:300), F4/80 (1:2000; detected by Opal 570 at 1:300) and CD138 (1:1200; detected by Opal 520 at 1:300). Counterstaining was performed using 4′,6-diamidino-2-phenylindole (Akoya Biosciences). Fluorochrome labeling was viewed under a Zeiss LSM780 confocal microscope and documented using the LSM780 software program.

### Western blotting

Pancreases were disrupted with TissueLyser LT (QIAGEN) and homogenized in RIPA buffer. The homogenates, which included 80 μg proteins, were subjected to SDS-PAGE (ATTO) and transferred onto Immobilon polyvinylidene difluoride membranes (Millipore). Blots were incubated with 5% nonfat dry milk in phosphate-buffered saline with 0.1% Tween 20 at room temperature for 1 h to block nonspecific binding, overnight at 4 °C with primary antibodies in blocking buffer, and finally with horseradish peroxidase conjugated secondary antibodies in blocking buffer at room temperature for 1 h. The membranes were developed with Chemi-Lumi One L and super (Nacalai Tesque), and analyzed in a WSE-6100H LuminoGraph1 (ATTO). Akt and actin were used as loading controls.

### Transmission electron microscopy

Anesthetized mice were fixed with 2% glutaraldehyde and 2% paraformaldehyde in 0.1 M phosphate buffer at pH 7.4. Pancreatic tissues were extracted after fixation. Slices of the fixed tissues were postfixed with 2% OsO4, dehydrated in ethanol and embedded in Epok 812 (Okenshoji Co.). Ultrathin sections were cut with an ultramicrotome (ultracut N or UC6: Leica). These sections were stained with uranyl acetate and lead citrate and examined on a Hitachi HT7700 or JEOL JEM-1230 electron microscope.

### Reverse transcriptase RT-PCR

Total RNA was isolated using an RNeasy Plus Universal Mini Kit (Qiagen). cDNA was synthesized using a ReverTra Ace qPCR RT Kit (Toyobo). For the detection of *Il6* mRNA, *Tnfα* and *Il1β*, a Thermal Cycler Dice Real Time PCR System III was used with SYBR Premix Ex Taq (Takara).

### Statistical analysis

Data in graphs are expressed as the mean ± standard error of the mean (SEM). Results were analyzed by an unpaired Student's *t*-test to compare two groups and a one-way analysis of variance (ANOVA) to compare multiple groups using the JMP Pro13 software program (SAS Institute Inc.). *P* values of < 0.05 were considered to indicate statistical significance.

## Supplementary Information

Supplementary Figures.
