# Supplementary material for: Cathepsin B and D deficiency in the mouse pancreas induces impaired autophagy and chronic pancreatitis
Source: Sci Rep. 2021 Mar 23;11:6596. doi: 10.1038/s41598-021-85898-9 (PMC7988038; doi:10.1038/s41598-021-85898-9)
Supplement: Supplementary file 1 — Supplementary Figures. [file 41598_2021_85898_MOESM1_ESM.pdf]

Cathepsin B and D deficiency in the mouse pancreas induces impaired autophagy and chronic pancreatitis

Hideaki Iwama[1,2], Sally Mehanna[3,7], Mai Imasaka[1], Shinsuke Hashidume[1], Hiroshi Nishiura[4], Ken-ichi Yamamura[3], Chigure Suzuki[5], Yasuo Uchiyama[6], Etsuro Hatano[2], Masaki Ohmuraya[1]

1. Department of Genetics, Hyogo College of Medicine, Nishinomiya, Hyogo, 663-8501, Japan
2. Department of Gastroenterological Surgery, Hyogo College of Medicine, Nishinomiya, Hyogo, 663-8501, Japan
3. Institute of Resource Development and Analysis, Kumamoto University, 2-2-1 Honjo, Chuo-ku, Kumamoto, 860-0811, Japan
4. Division of Functional Pathology, Department of Pathology, Hyogo College of Medicine, Nishinomiya, Hyogo, 663-8501, Japan
5. Department of Pharmacology, Juntendo University Graduate School of Medicine, 2-1-1 Hongo, Bunkyo-ku, Tokyo, 113-8421, Japan
6. Department of Cell Biology and Neuroscience, Juntendo University Graduate School of Medicine, 2-1-1 Hongo, Bunkyo-ku, Tokyo, 113-8421, Japan
7. Present Address:Department of Veterinary Hygiene and Management, Faculty of Veterinary Medicine, Cairo University, Giza, 12211, Egypt

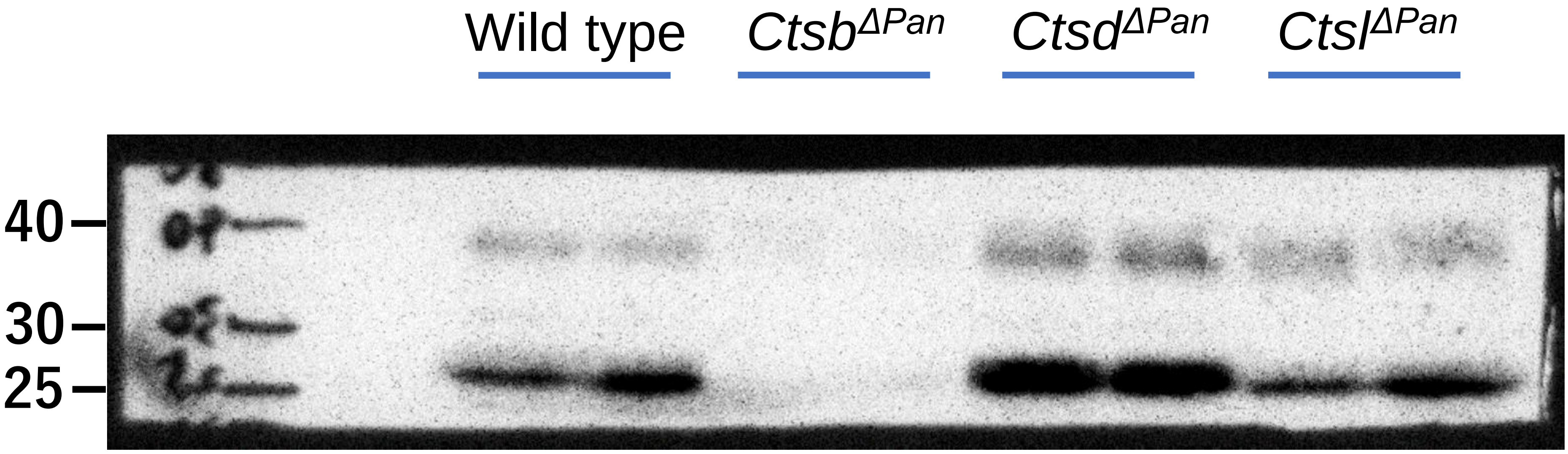

Primary antibody:
Ctsb (R&B AF965, 1:2500)

Chemiluminescent reagent:
Chemi-lumi One L (ex 5min)

Gel : e-PAEL®(5-20%)

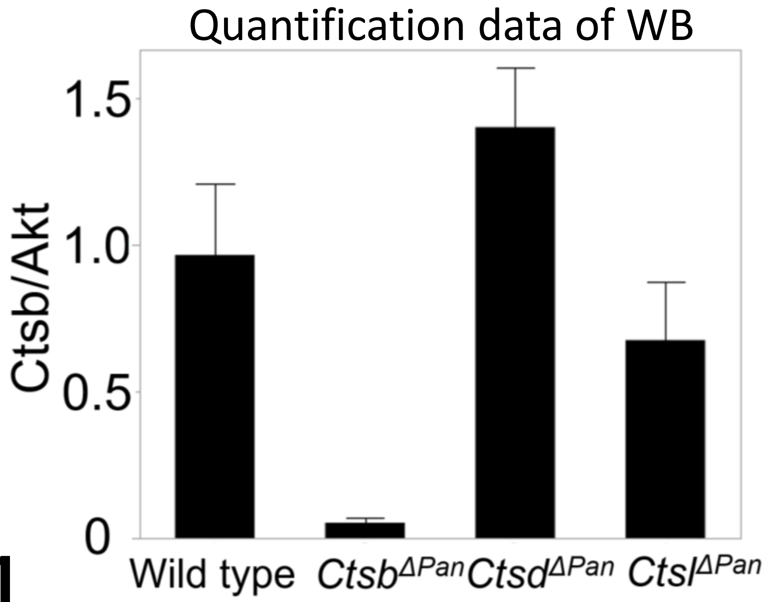

P<0.05

Figure 1

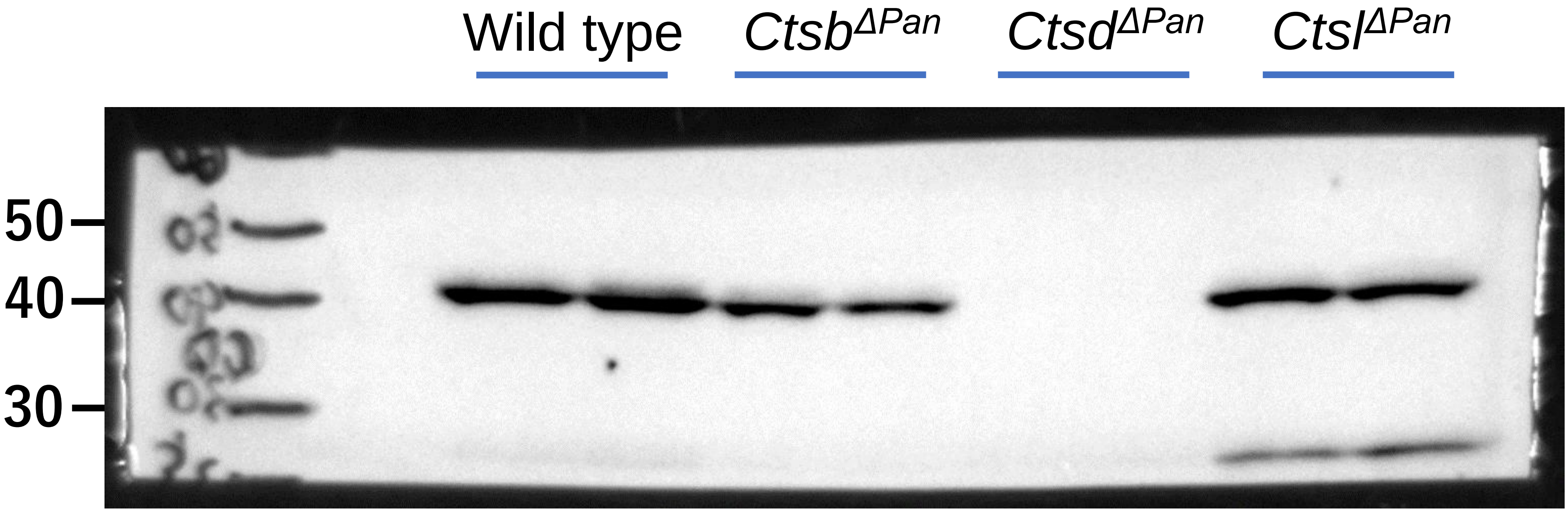

Primary antibody:
Ctsd (santa cruz sc-6486,1:500)

Chemiluminescent reagent:
Chemi-lumi One L (ex 5min)

Gel : e-PAEL®(5-20%)

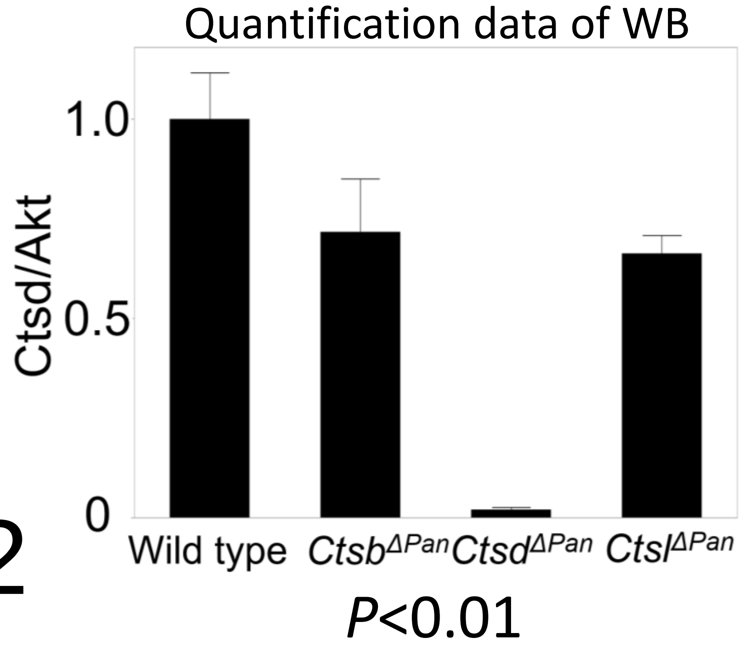

Figure 2

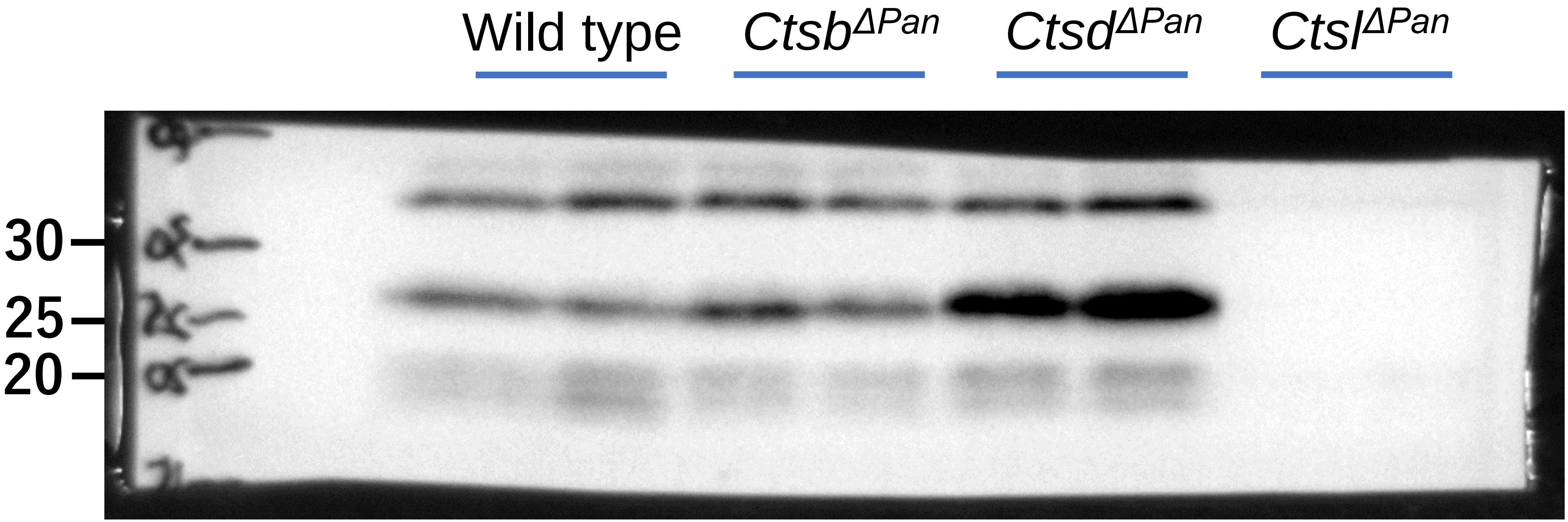

Primary antibody:
Ctsl (R&B AF1515, 1:2000)

Chemiluminescent reagent:
Chemi-lumi One L (ex 5min)

Gel : e-PAEL®(5-20%)

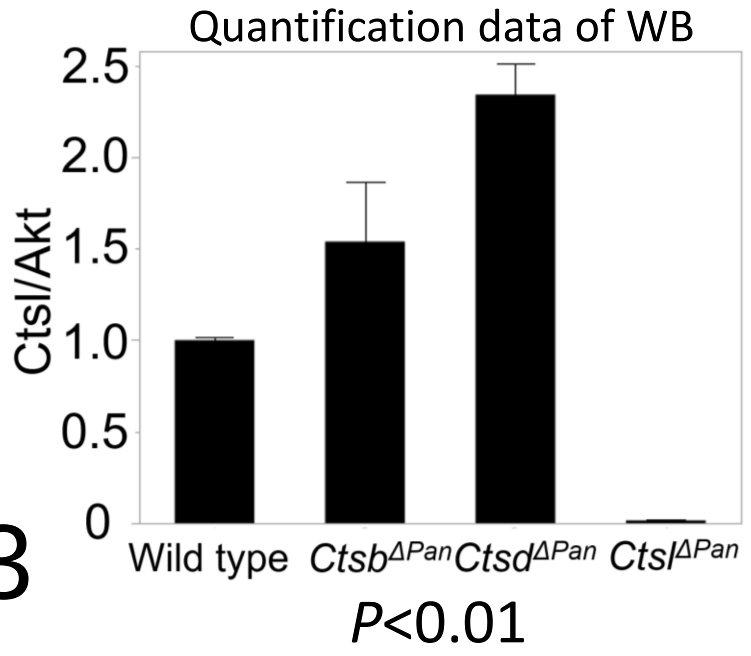

Figure 3

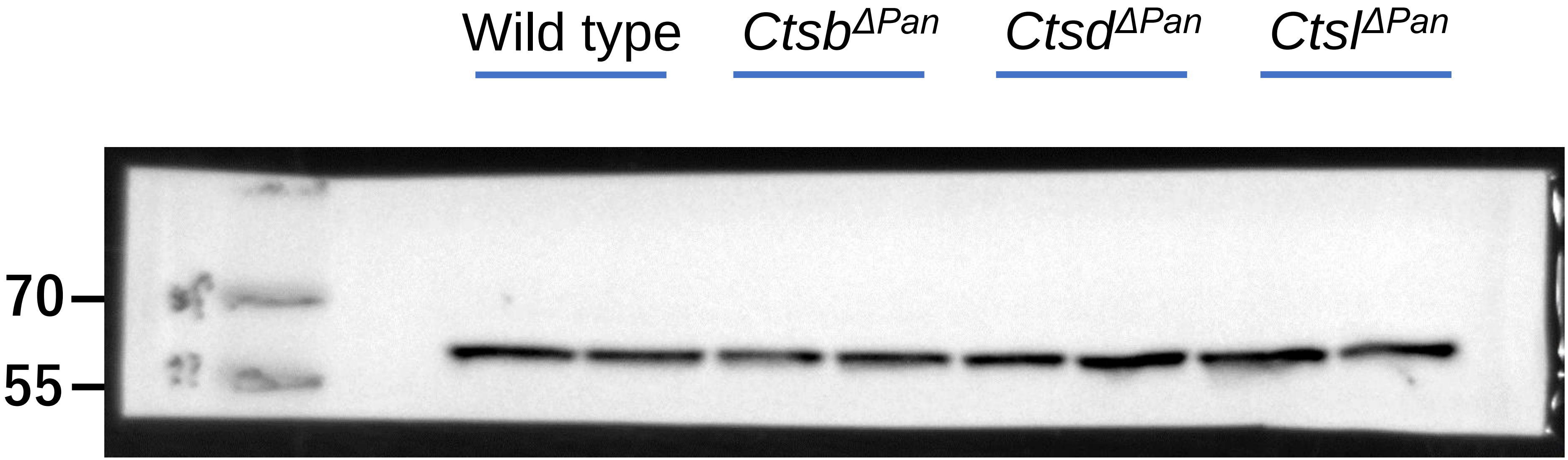

Primary antibody : Akt (CST 9272, 1:1000)

Chemiluminescent reagent : Chemi-lumi One L (ex 2min)

Gel : e-PAEL®(5-20%)

Figure 4

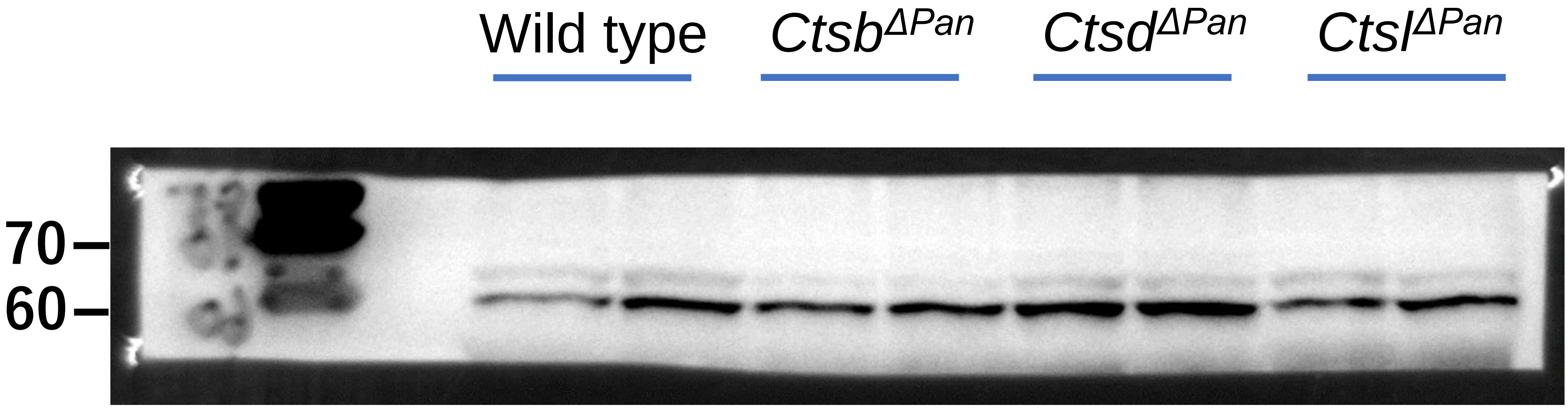

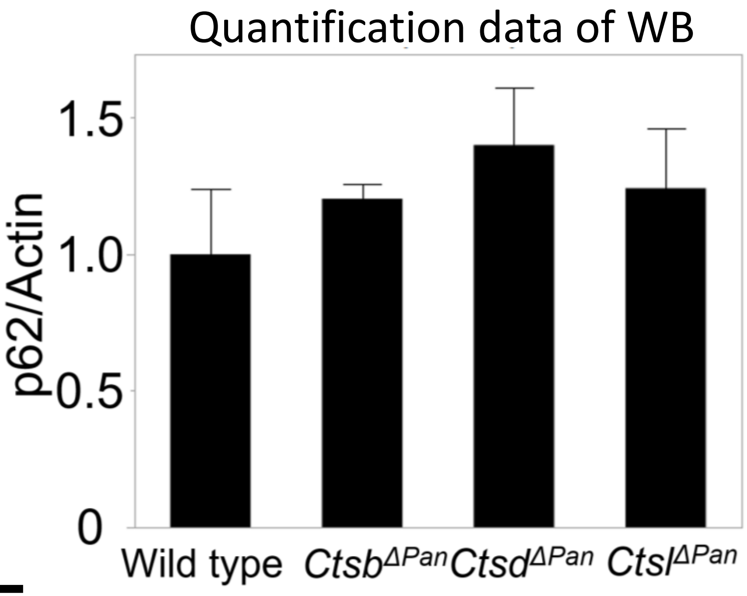

No significant difference

Primary antibody:
p62 (MBL PM045, 1:5000)

Chemiluminescent reagent:
Chemi-lumi One super (ex 1min)

Gel : e-PAEL®(5-20%)

Figure 5

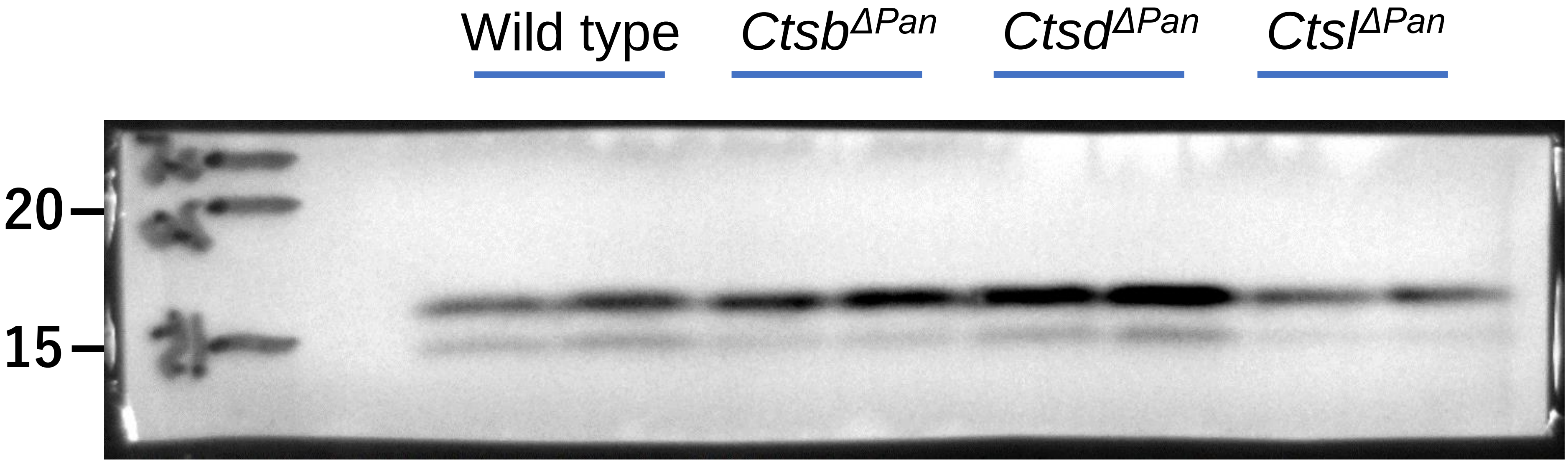

Primary antibody:
LC3 (CST 2775, 1:1000)

Chemiluminescent reagent:
Chemi-lumi One super (ex 3min)

Gel : e-PAEL®(5-20%)

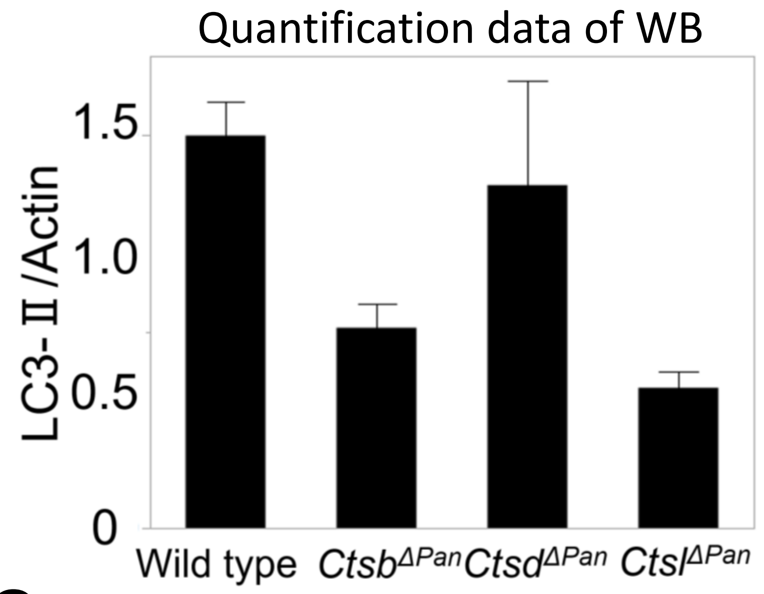

Figure 6

No significant difference

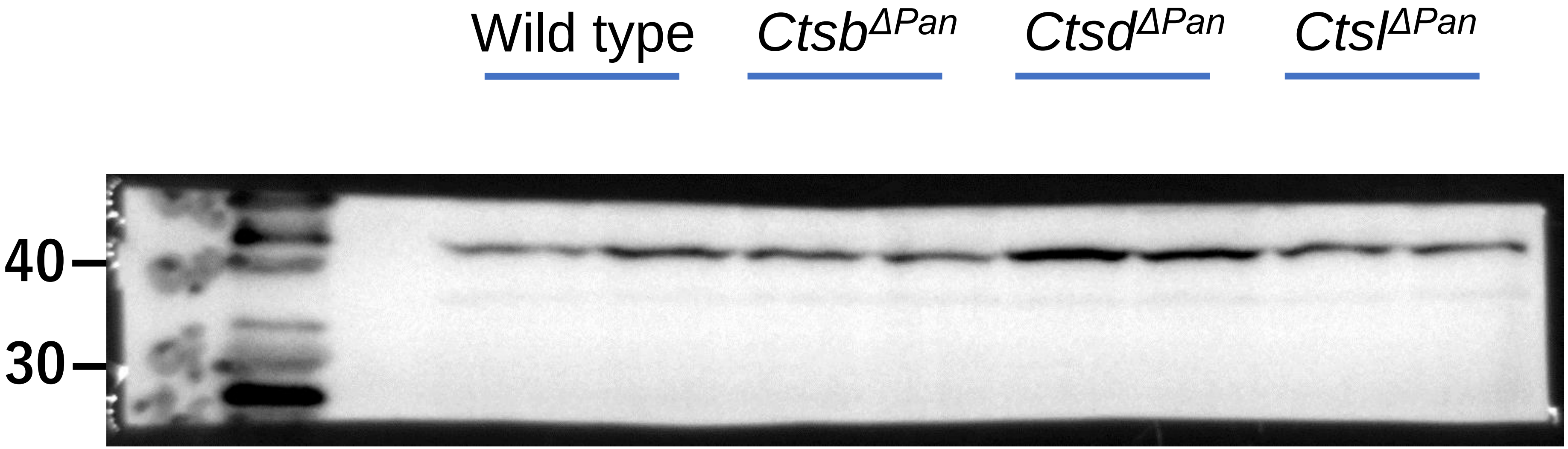

Primary antibody : Actin (Sigma A5060, 1:2000)

Chemiluminescent reagent : Chemi-lumi One super (ex 3min)

Gel : e-PAEL®(5-20%)

Figure 7

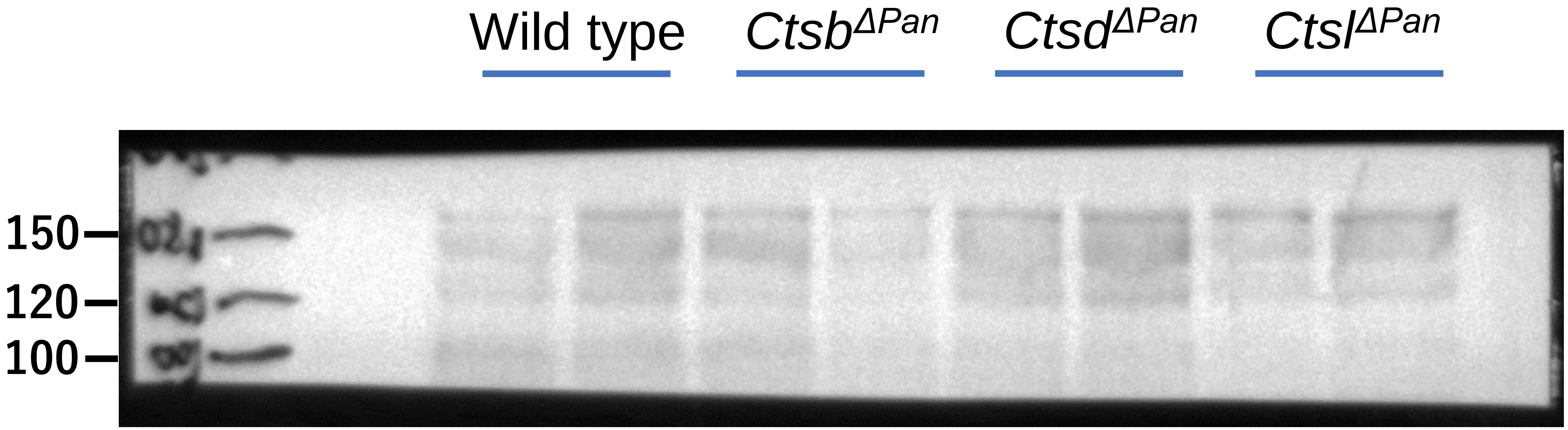

Primary antibody:
ULK1 (Sigma A7481, 1:1000)

Chemiluminescent reagent:
Chemi-lumi One L (ex 5min)

Gel : e-PAEL®(5-20%)

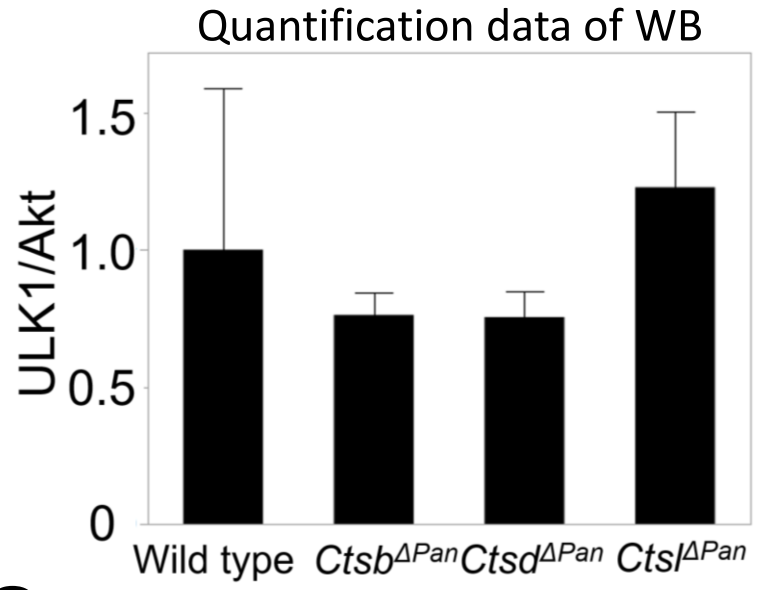

Figure 8

No significant difference

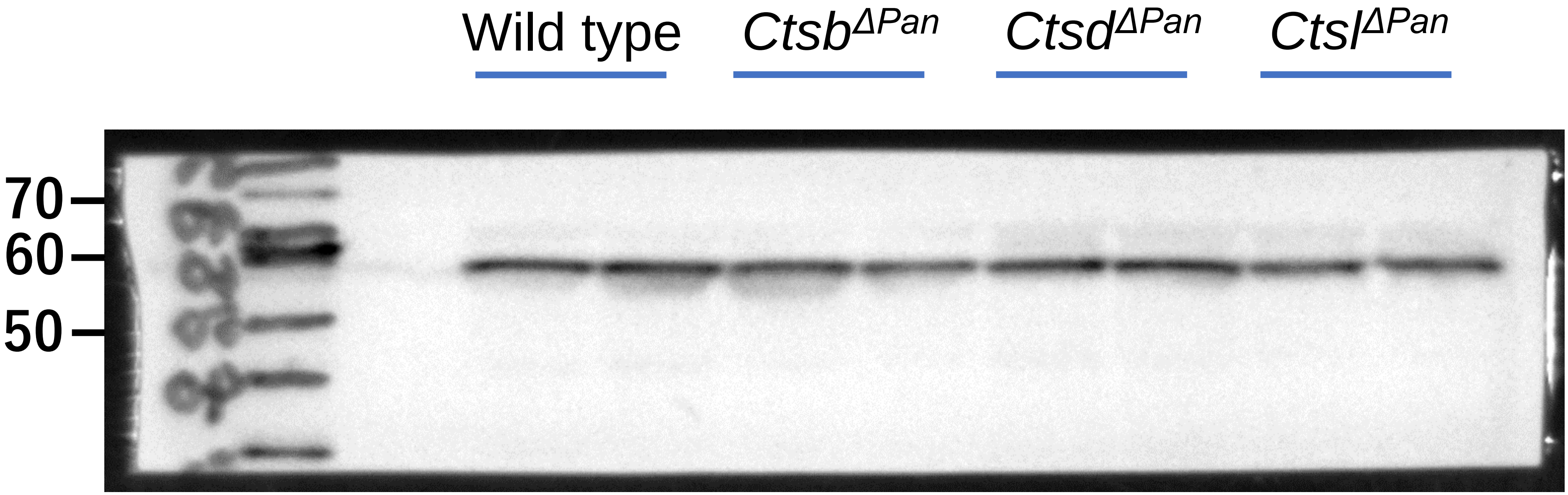

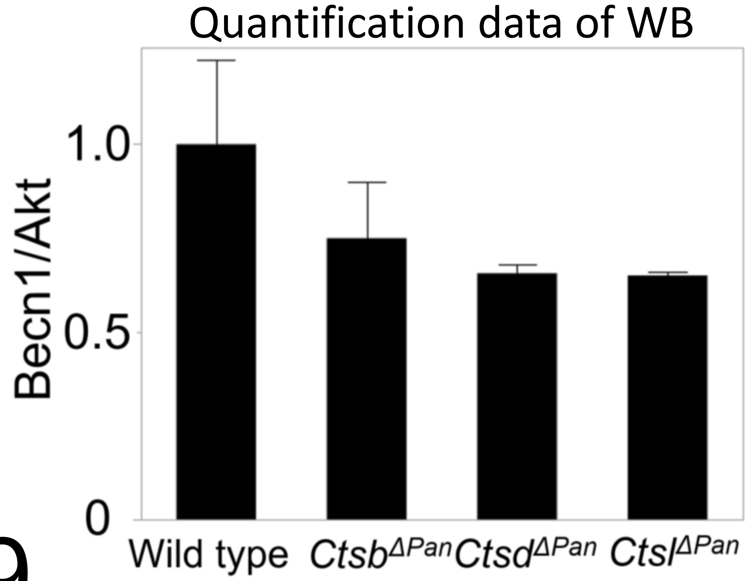

No significant difference

Primary antibody:
Becn1 (santa cruz 11427, 1:1000)

Chemiluminescent reagent:
Chemi-lumi One L (ex 5min)

Gel : e-PAEL®(5-20%)

Figure 9

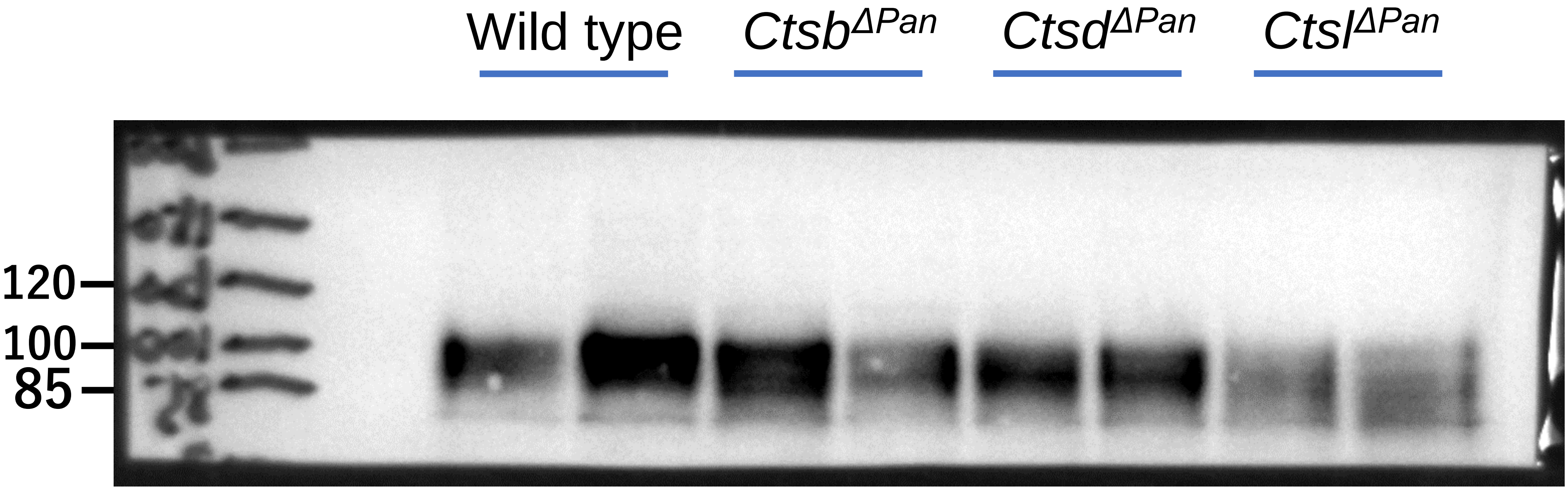

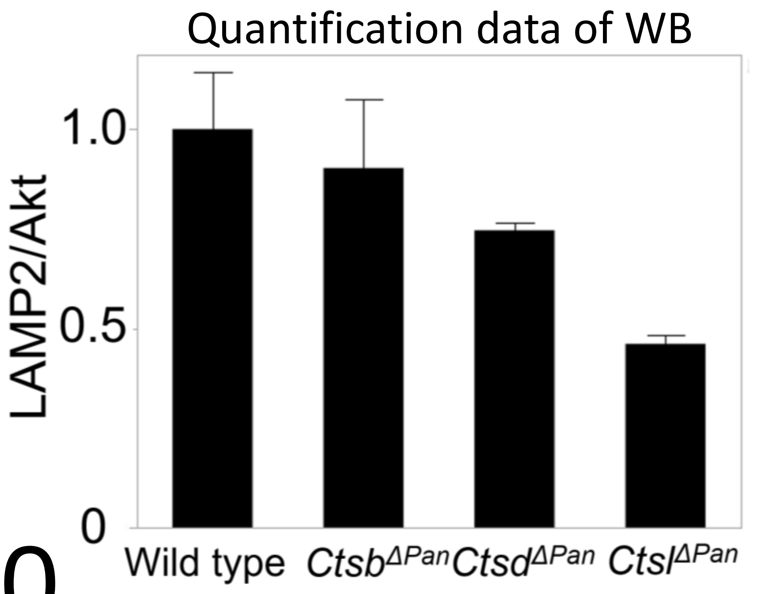

Primary antibody:
LAMP2 (Sigma L0668, 1:1000)

Chemiluminescent reagent:
Chemi-lumi One super (ex 1min)

Gel : e-PAEL®(5-20%)

Figure 10

No significant difference

Wild type $Ctsb^{\Delta Pan}$ $Ctsd^{\Delta Pan}$ $Ctsl^{\Delta Pan}$

25

20

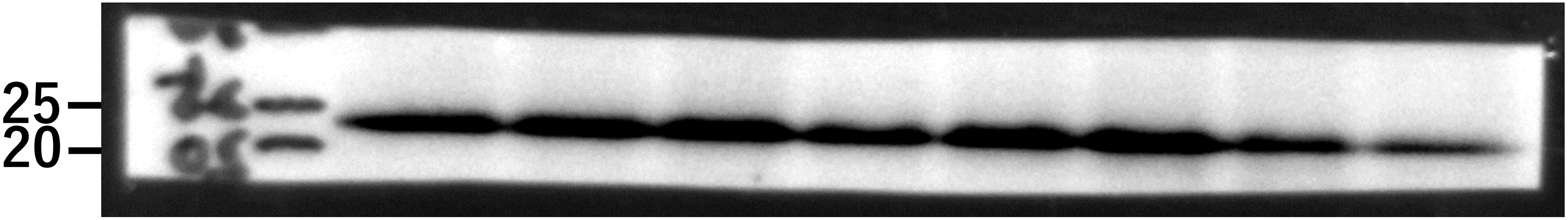

Primary antibody:
Rab7 (Sigma R8779, 1:1000)

Chemiluminescent reagent:
Chemi-lumi One L (ex 2min)

Gel : e-PAEL®(5-20%)

Quantification data of WB

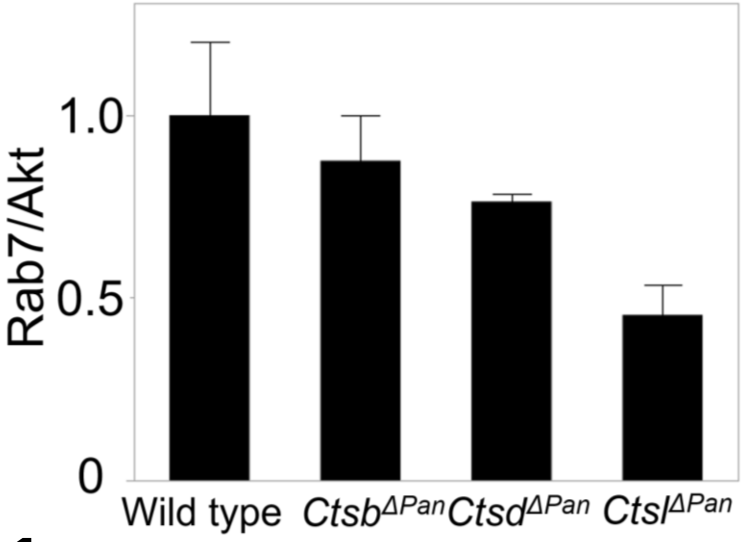

No significant difference

Figure 11

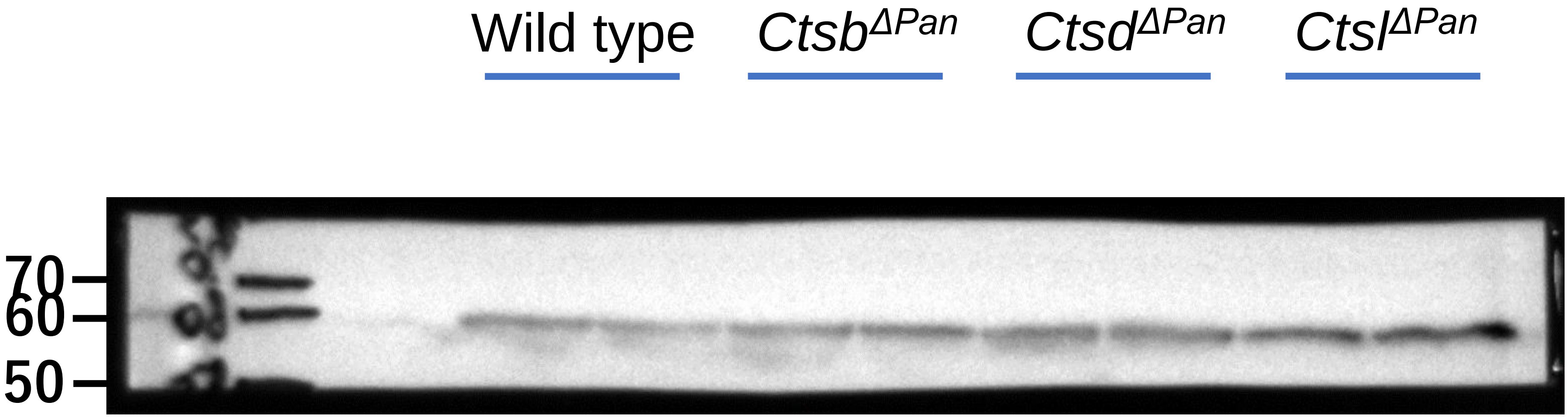

Primary antibody : Akt (CST 9272, 1:1000)

Chemiluminescent reagent : Chemi-lumi One L (ex 5min)

Gel : e-PAEL®(5-20%)

Figure 12

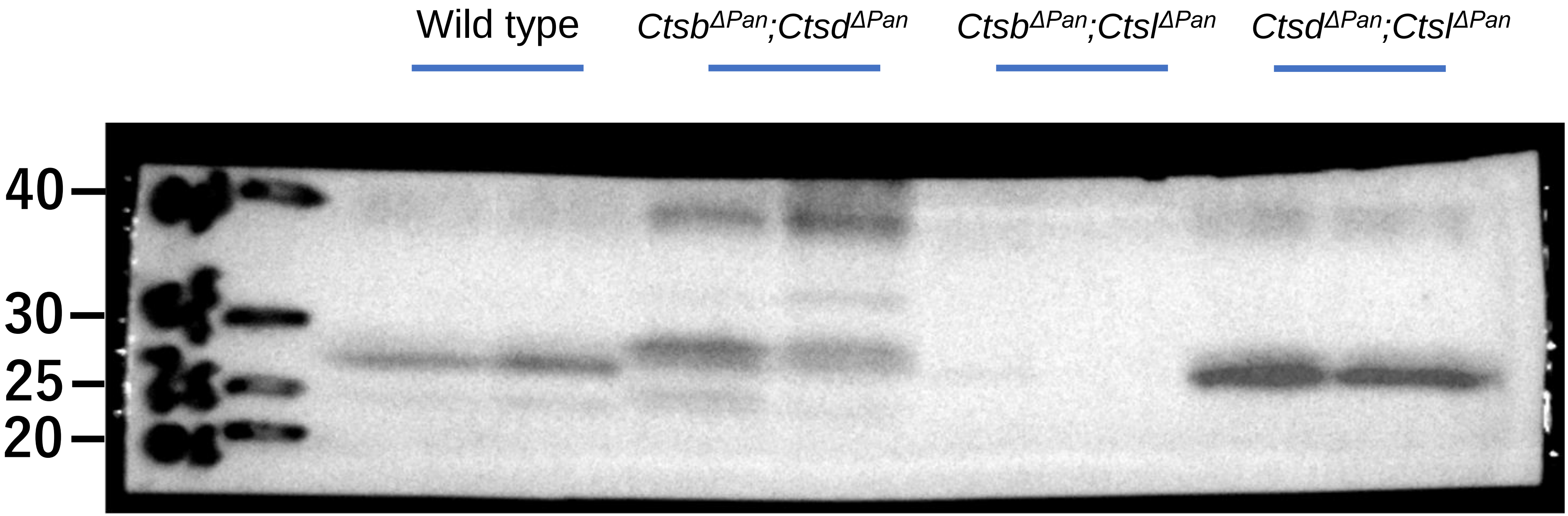

Primary antibody :
Ctsb (R&B AF965, 1:2500)

Chemiluminescent reagent :
Chemi-lumi One L (ex 5min)

Gel : e-PAEL®(5-20%)

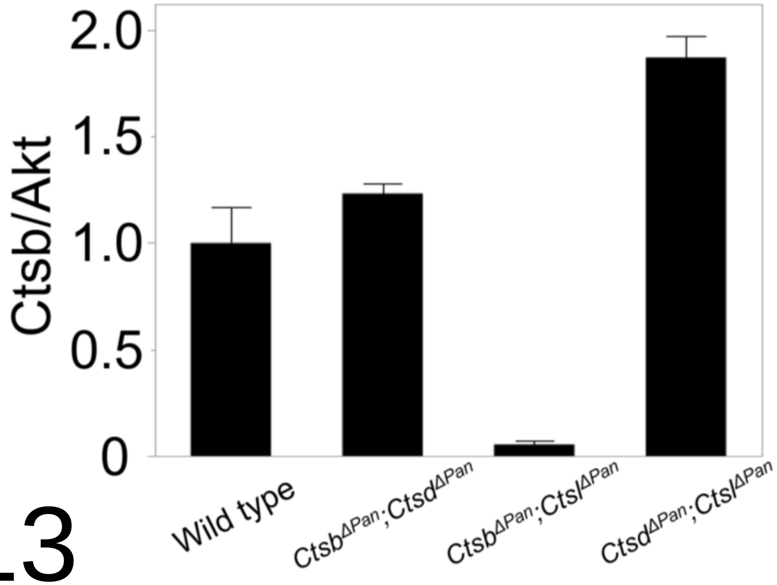

P<0.01

Figure 13

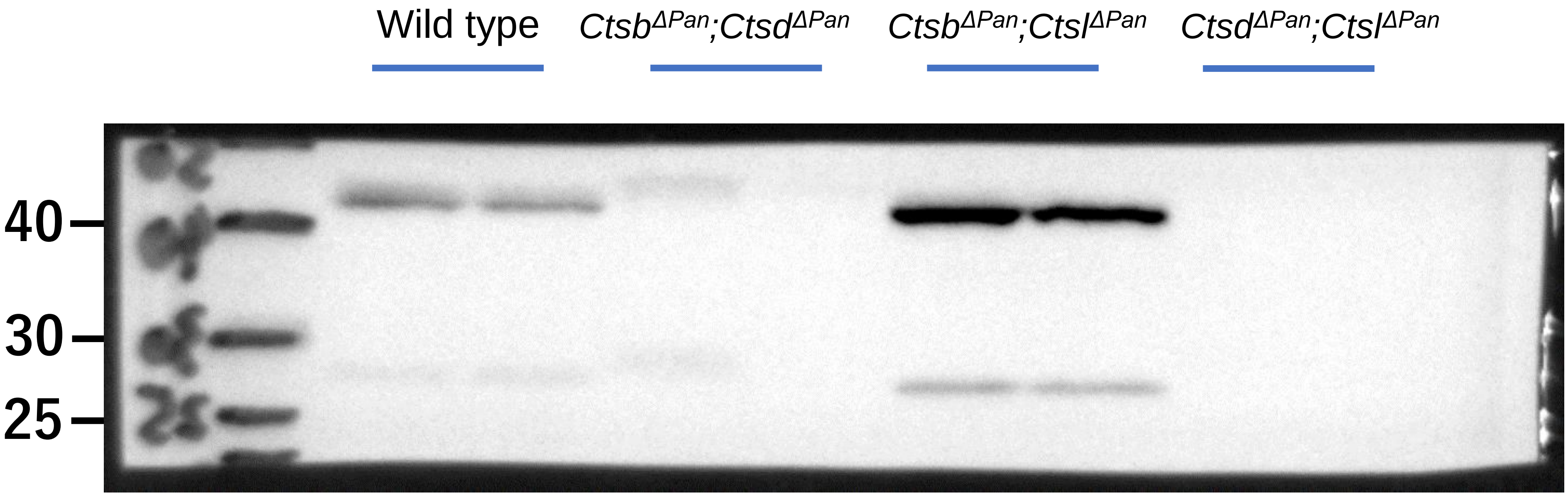

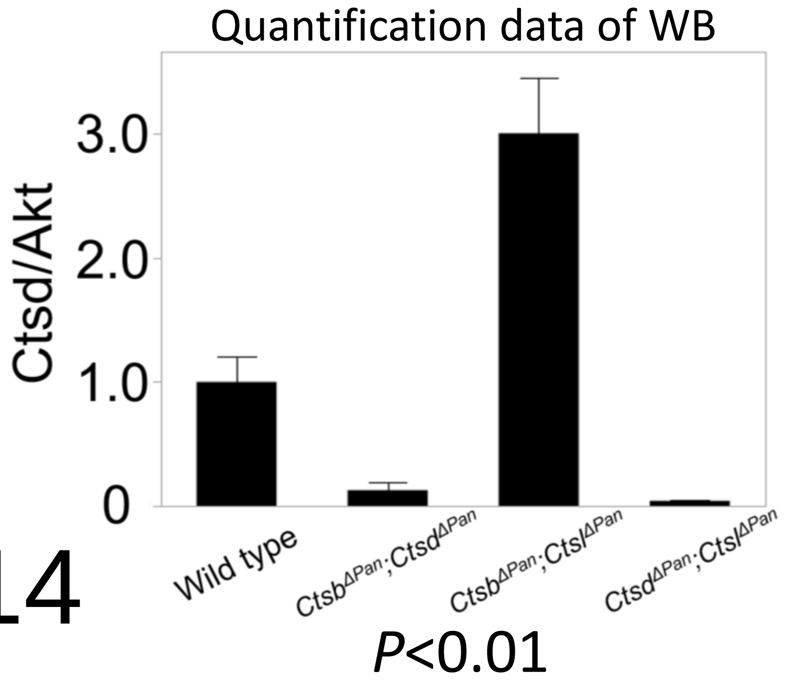

Primary antibody :
Ctsd (santa cruz sc-6486,1:500)

Chemiluminescent reagent :
Chemi-lumi One L (ex 5min)

Gel : e-PAEL®(5-20%)

Figure 14

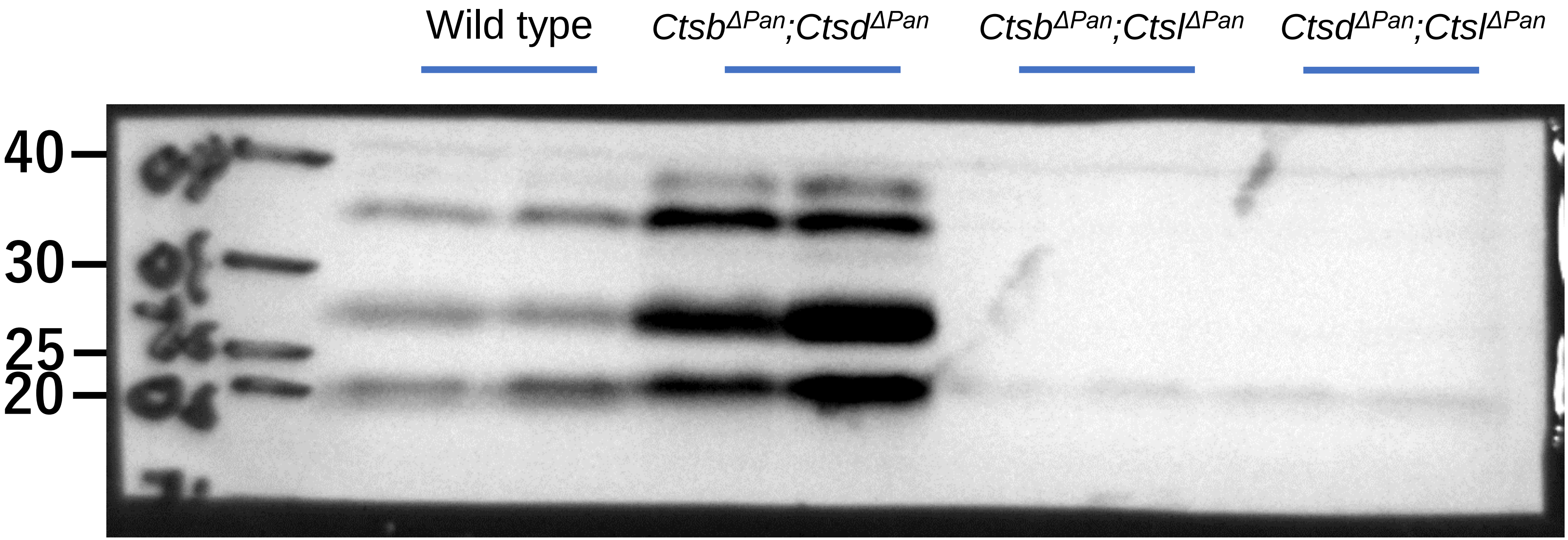

Quantification data of WB

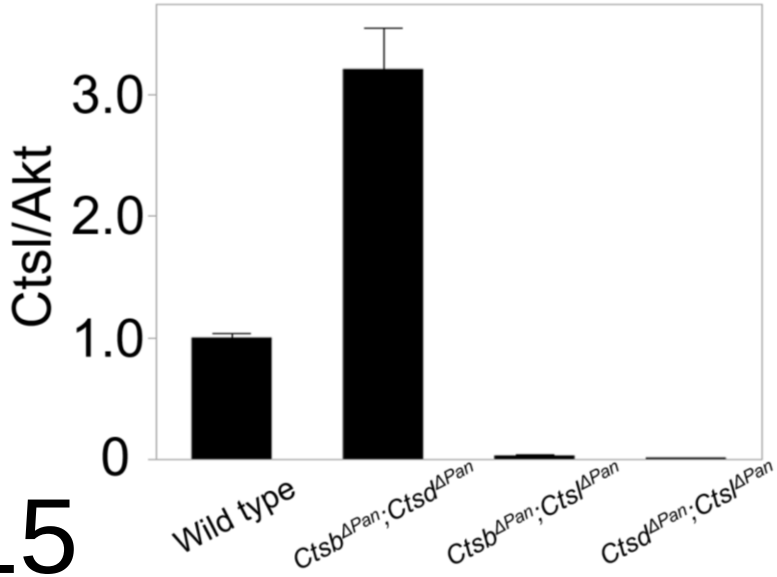

P<0.01

Primary antibody :
Ctsl (R&B AF1515, 1:2000)

Chemiluminescent reagent :
Chemi-lumi One L (ex 5min)

Gel : e-PAEL®(5-20%)

Figure 15

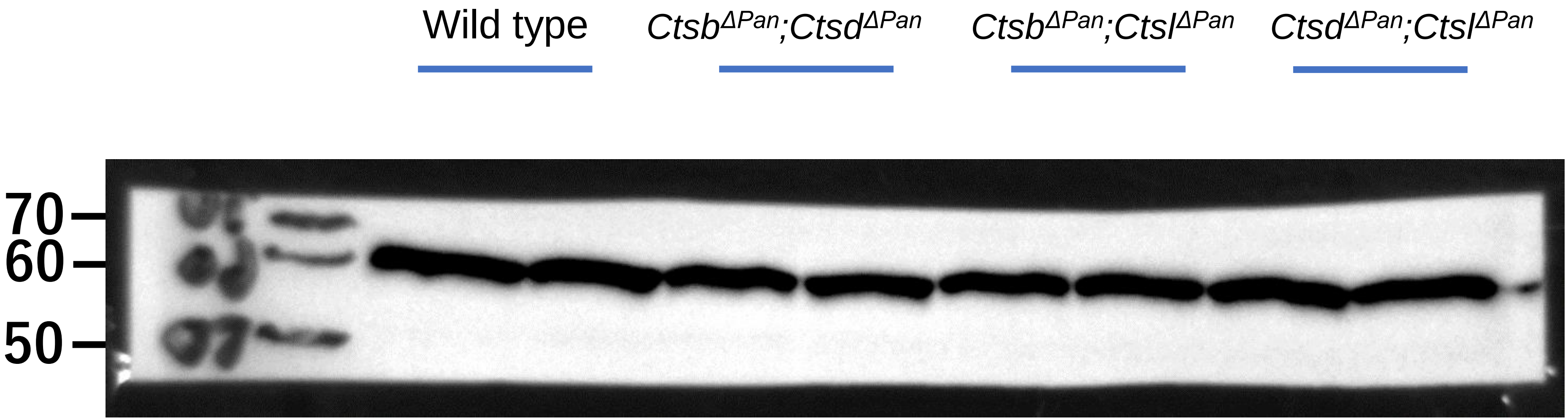

Primary antibody : Akt (CST 9272, 1:1000)

Chemiluminescent reagent : Chemi-lumi One L (ex 1min)

Gel : e-PAEL®(5-20%)

Figure 16

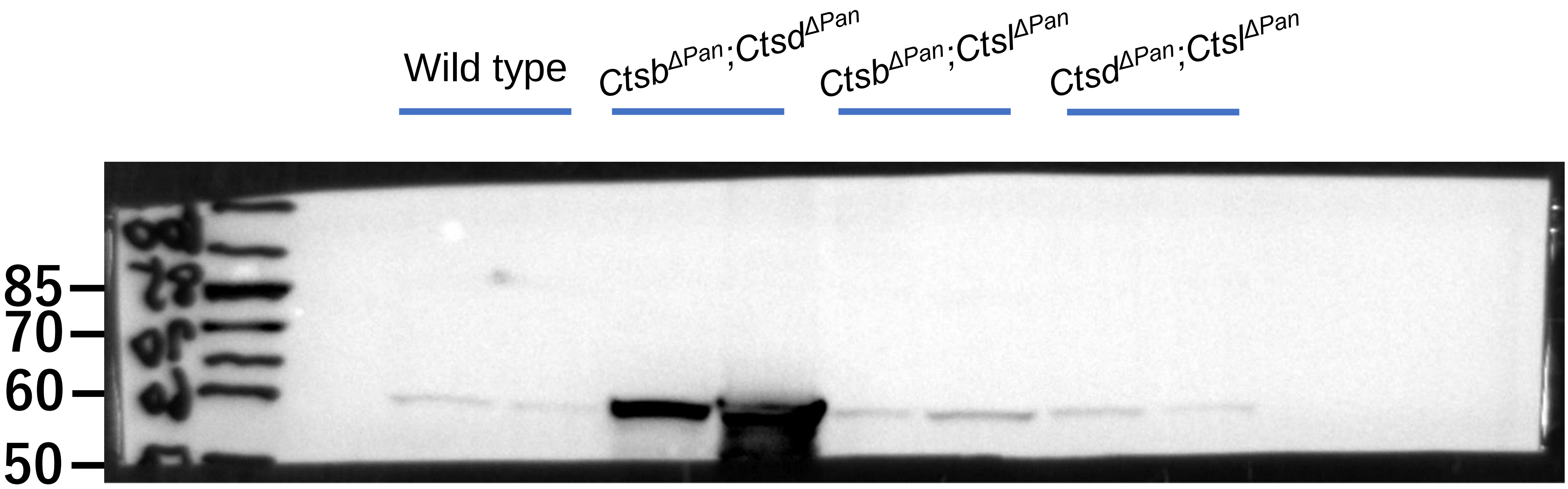

Primary antibody :
p62 (MBL PM045, 1:5000)

Chemiluminescent reagent :
Chemi-lumi One L (ex 1min)

Gel : e-PAEL®(5-20%)

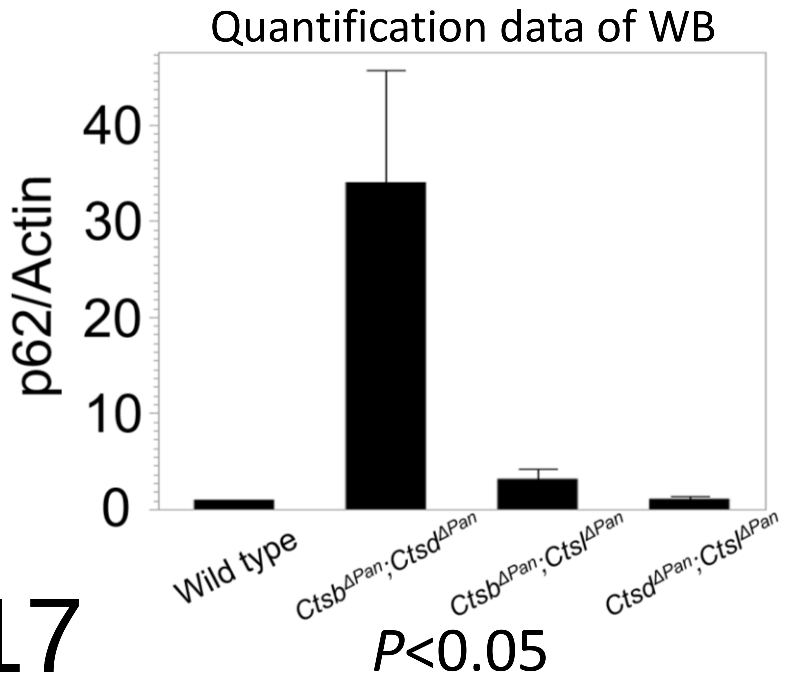

Figure 17

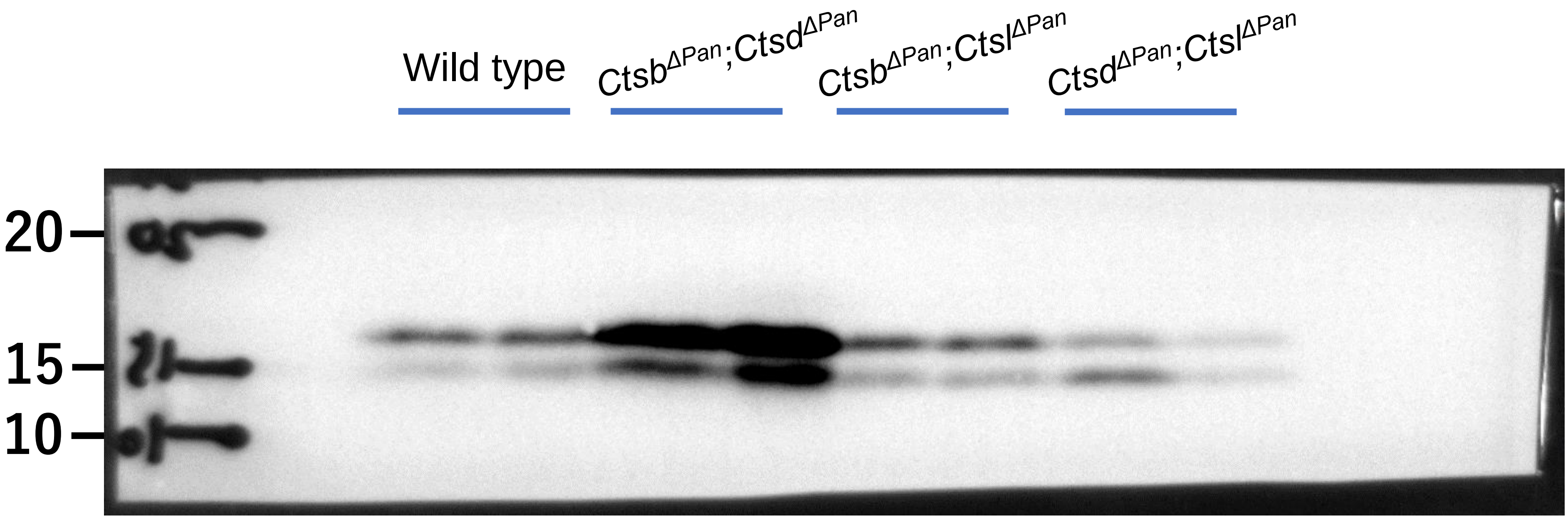

Primary antibody :
LC3 (CST 2775, 1:1000)

Chemiluminescent reagent :
Chemi-lumi One L (ex 5min)

Gel : e-PAEL®(5-20%)

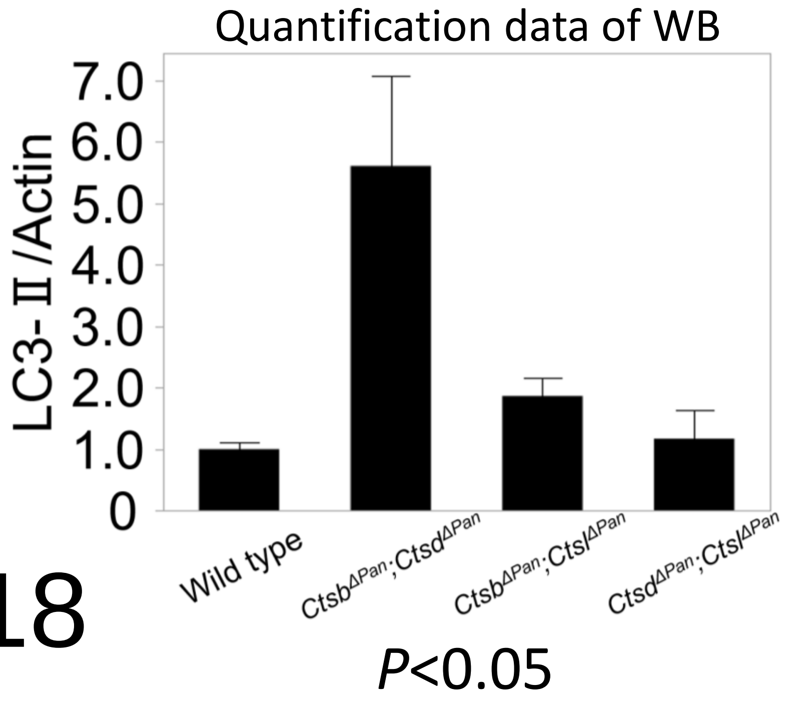

Figure 18

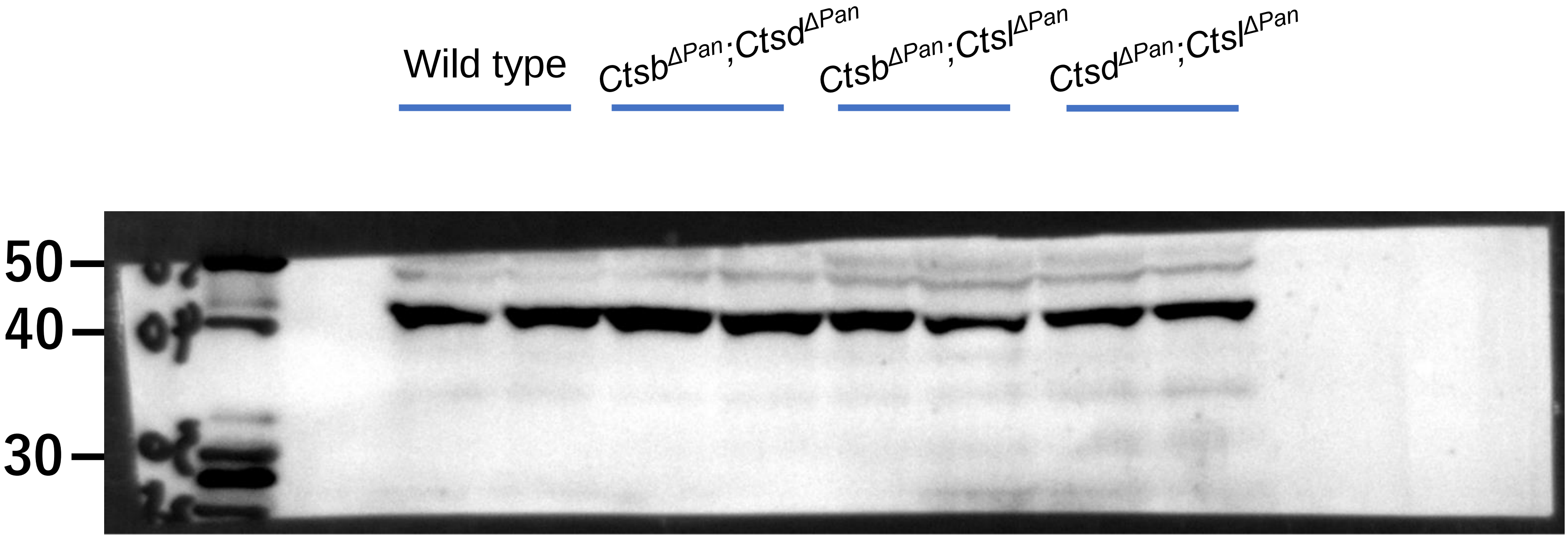

Primary antibody : Actin (Sigma A5060,1:2000)

Chemiluminescent reagent : Chemi-lumi One L (ex 3min)

Gel : e-PAEL®(5-20%)

Figure 19

Wild type | $Ctsb^{\Delta Pan}$;$Ctsd^{\Delta Pan}$ | $Ctsb^{\Delta Pan}$;$Ctsl^{\Delta Pan}$ | $Ctsd^{\Delta Pan}$;$Ctsl^{\Delta Pan}$

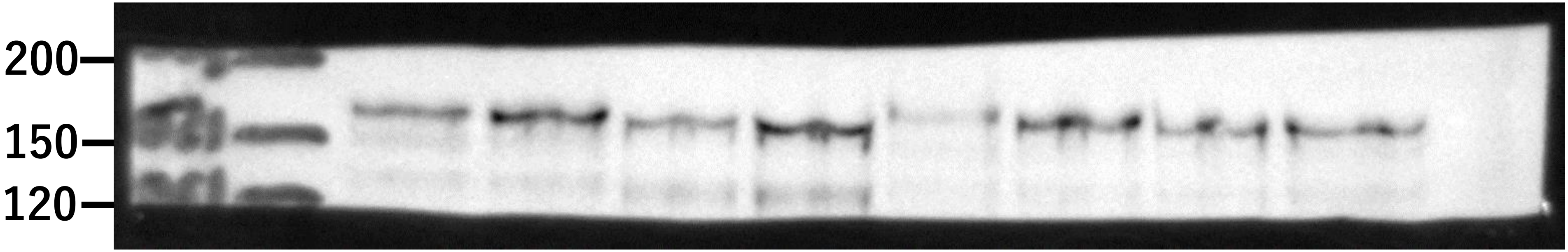

Primary antibody :
ULK1 (Sigma A7481, 1:1000)

Chemiluminescent reagent :
Chemi-lumi One L (ex 5min)

Gel : e-PAEL®(5-20%)

Quantification data of WB

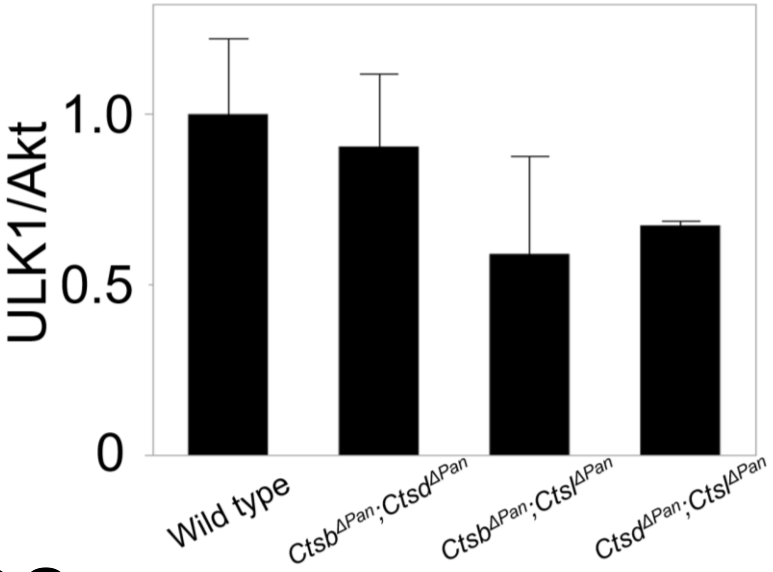

Figure 20

No significant difference

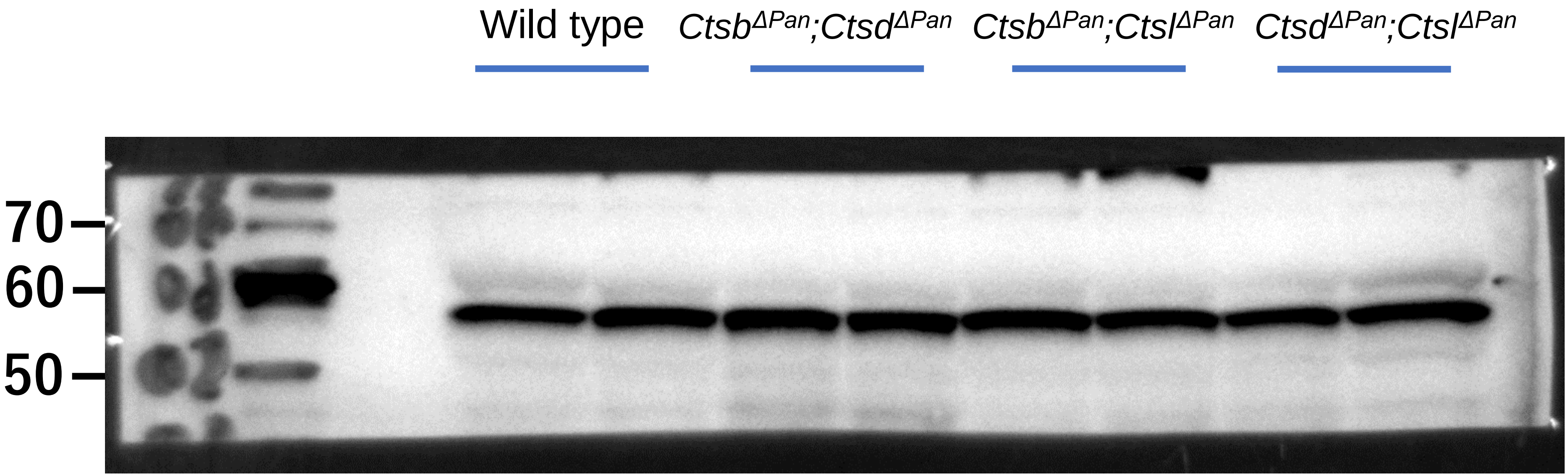

Primary antibody :
Becn1 (santa cruz 11427,1:1000)

Chemiluminescent reagent :
Chemi-lumi One super (ex 2min)

Gel : e-PAEL®(5-20%)

Quantification data of WB

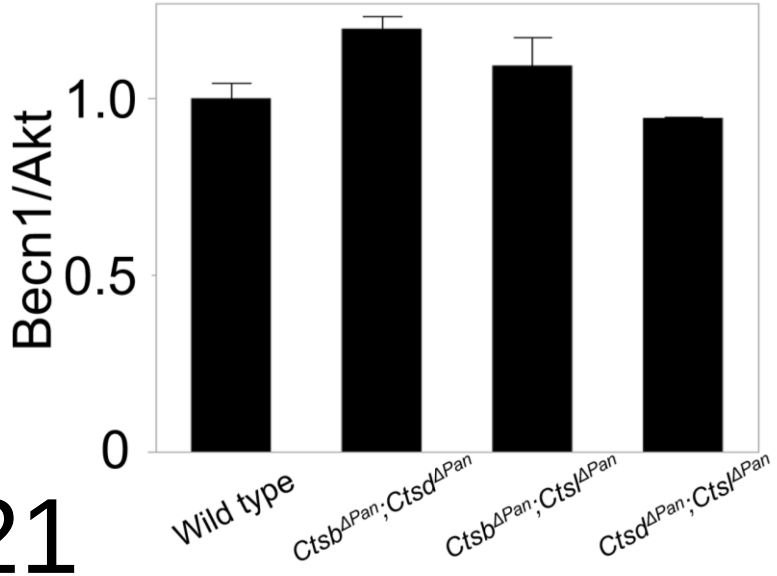

No significant difference

Figure 21

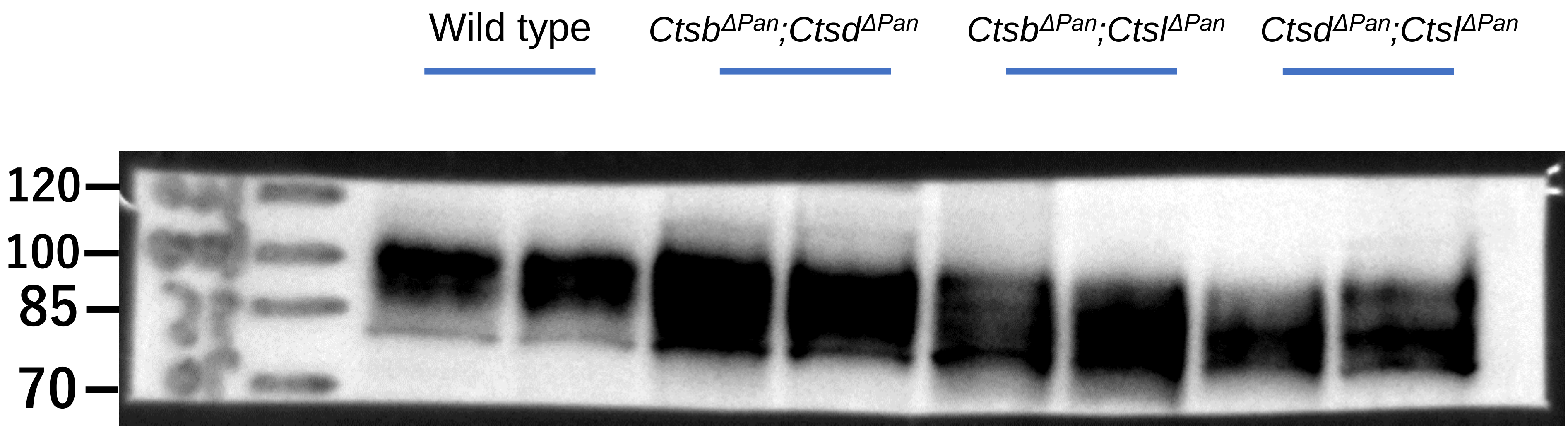

Primary antibody :
LAMP2 (Sigma L0668, 1:1000)

Chemiluminescent reagent :
Chemi-lumi One super (ex 3min)

Gel : e-PAEL®(5-20%)

Quantification data of WB

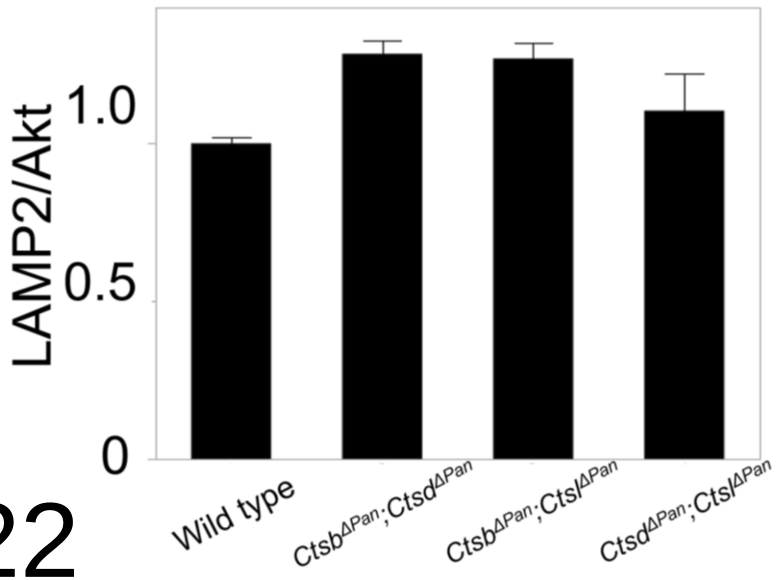

No significant difference

Figure 22

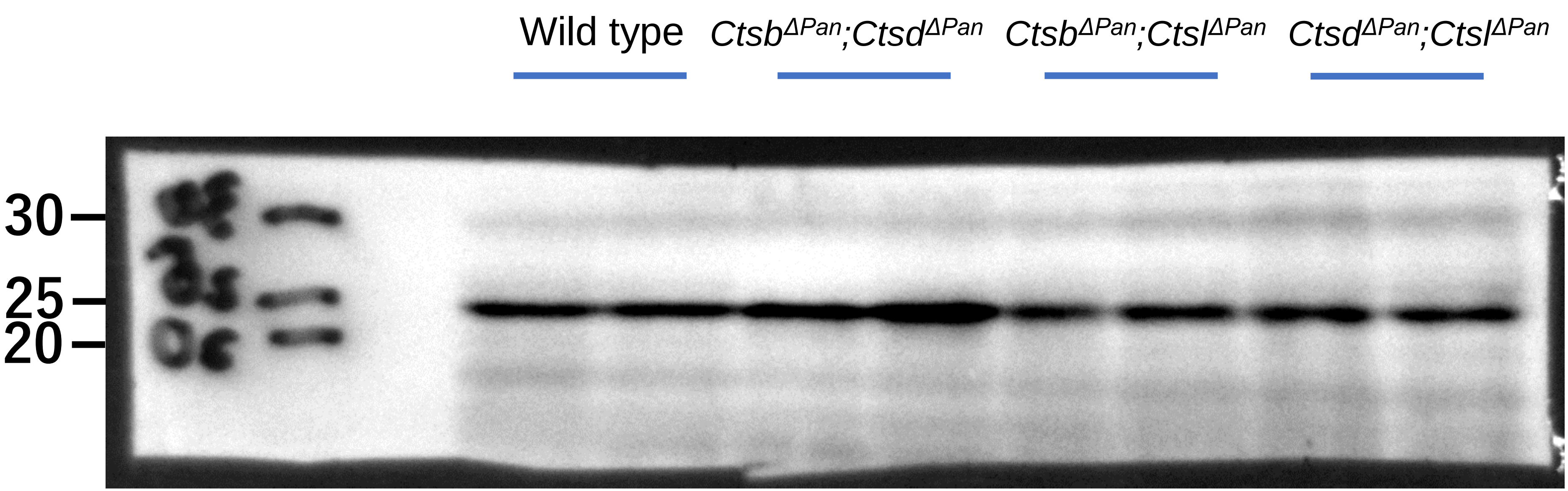

Primary antibody :
Rab7 (Sigma R8779, 1:1000)

Chemiluminescent reagent :
Chemi-lumi One super (ex 5min)

Gel : e-PAEL®(5-20%)

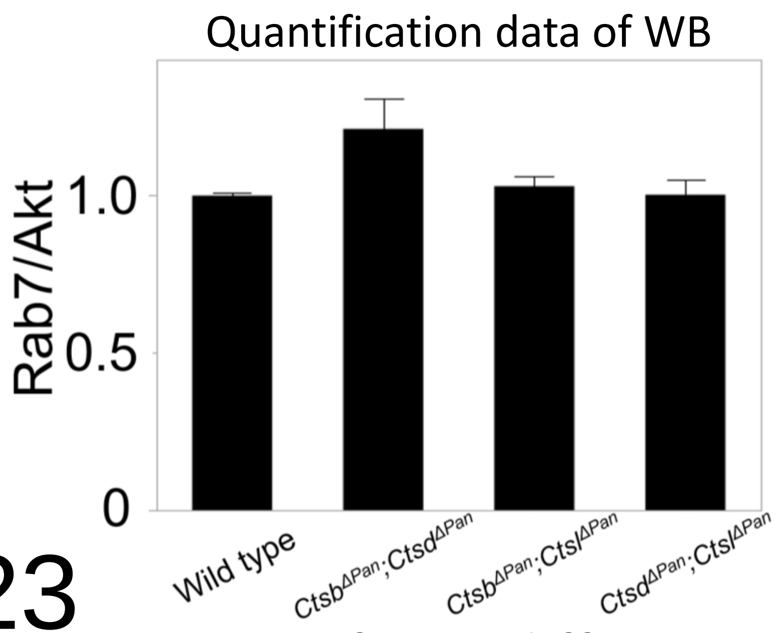

Figure 23

No significant difference

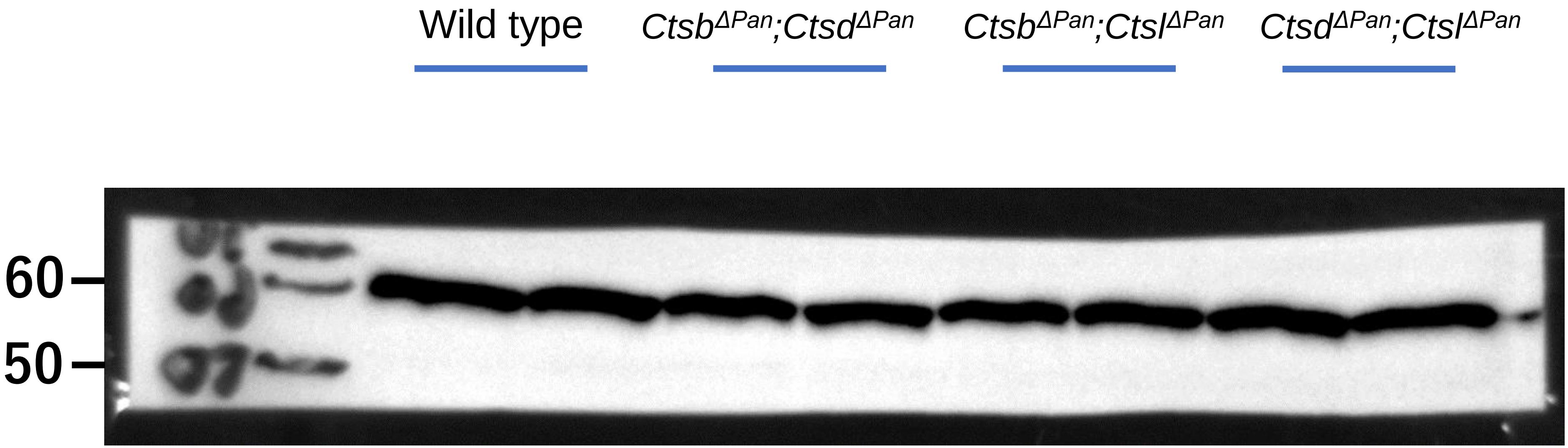

Primary antibody : Akt (CST 9272, 1:1000)

Chemiluminescent reagent : Chemi-lumi One L (ex 1min)

Gel : e-PAEL®(5-20%)

Figure 24
